# Identifying Eating Disorders in Adolescents and Adults Living With Higher Weight: An Updated Systematic Review of Questionnaire Diagnostic Accuracy

**DOI:** 10.1002/eat.70132

**Published:** 2026-05-20

**Authors:** Eve T. House, Caitlin M. McMaster, Natalie B. Lister, Isabelle R. Jardine, Sasha J. Lorien, Anna Lene Seidler, Hiba Jebeile

**Affiliations:** ^1^ Sydney Medical School, Faculty of Medicine and Health The University of Sydney Westmead New South Wales Australia; ^2^ Charles Perkins Centre The University of Sydney Camperdown New South Wales Australia; ^3^ Sydney School of Health Sciences, Faculty of Medicine and Health The University of Sydney Camperdown New South Wales Australia; ^4^ South Western Sydney Local Health District Liverpool New South Wales Australia; ^5^ Ingham Institute for Applied Medical Research Liverpool New South Wales Australia; ^6^ Eating Disorder and Nutrition Research Group (ENRG), Translational Health Research Institute, Faculty of Medicine Western Sydney University Campbelltown New South Wales Australia; ^7^ Department of Child and Adolescent Psychiatry University Medical Centre Rostock Rostock Germany; ^8^ German Centre for Child and Adolescent Health (DZKJ), Partner Site Greifswald/Rostock Rostock Germany

**Keywords:** assessment, diagnostic accuracy, disordered eating, eating disorders, obesity, questionnaires, screening

## Abstract

**Objective:**

To update the evidence regarding the diagnostic accuracy of eating disorder (ED) questionnaires in adolescents and adults with higher weight.

**Method:**

Five databases were systematically searched from 2020 to November 2025 (CRD420251186115). Included studies reported on the diagnostic accuracy of self‐report questionnaires against a clinical interview to identify EDs and disordered eating behaviors (DEBs) in adolescents and adults with higher weight. Narrative synthesis was conducted, and findings from new studies were synthesized alongside previously identified studies.

**Results:**

Thirty‐two studies (5 new) were included, reporting on the diagnostic accuracy of 13 questionnaires in adults and 5 in adolescents. The diagnostic accuracy of questionnaires was examined to identify any ED (5 questionnaires in adults, 0 adolescents), binge‐eating disorder (8 adult, 2 adolescent), DEBs (e.g., binge eating, purging) (4 adult, 1 adolescent), loss‐of‐control eating (1 adult, 2 adolescent), bulimia nervosa, atypical anorexia nervosa, purging disorder, and night eating syndrome (each *n* = 1 adult, 0 adolescent). The Eating Disorder Examination Questionnaire (7 studies; sensitivity 0.16–0.88; specificity 0.54–1.0), Binge Eating Scale (6 studies; sensitivity 0.37–0.98; specificity 0.48–0.96), and Questionnaire on Eating and Weight Patterns (6 studies; sensitivity 0.07–1.0; specificity 0.0–1.0) were most used.

**Discussion:**

Progress in evaluation of the diagnostic accuracy of ED questionnaires in people with higher weight has been limited. There remains a lack of evidence regarding the diagnostic accuracy of questionnaires in adolescents and a lack of sufficiently sensitive questionnaires to identify EDs other than binge‐eating disorder. Assessing the diagnostic accuracy of questionnaires in people with higher weight remains a research priority.

## Summary

- Eating disorders and higher weight commonly co‐occur; eating disorder screening and assessment is recommended as part of care for people seeking support with weight loss; however, recommendations on which tools to use are lacking.
- Thirty‐two studies have been identified that report on the diagnostic accuracy of self‐reported eating disorder questionnaires, with the majority focused on identifying binge‐eating disorder or binge‐eating behaviors.
- To date, the diagnostic accuracy of the Binge Eating Scale has been most extensively assessed to identify binge‐eating disorder in adults, generally demonstrating good sensitivity.
- Diagnostic accuracy studies of eating disorder questionnaires for adolescents with higher weight are lacking.
- Assessment of the diagnostic accuracy of existing questionnaires or development of tailored questionnaires to identify restrictive eating disorders in adolescents and adults with higher weight is needed.

## 1 Introduction

Eating disorders (EDs) and higher weight can co‐occur. People living with higher weight, including clinical obesity (Rubino et al. 2025), may be at increased risk of developing EDs due to shared risk and maintenance factors (Camacho‐Barcia et al. 2024; da Luz et al. 2018). The 12‐month and lifetime prevalence of bulimia nervosa (BN), binge‐eating disorder (BED) and recurrent binge‐eating behaviors is higher among people with higher weight than those with lower weight (Duncan et al. 2017) and adolescents with probable EDs (including, but not limited to, BED, BN, atypical anorexia nervosa (AAN)) are more likely to be living with higher weight than those without these disorders (Mitchison et al. 2020). It is estimated that 14% of people presenting for obesity treatment have BED, and 1% have BN (Melville et al. 2025). However, the true prevalence of EDs, particularly restrictive EDs, among people living with higher weight may be higher. EDs may be under‐recognized in this population, due to a lack of awareness that all types of ED can present in people across the weight spectrum. This can lead to patients with higher weight experiencing delays in accessing ED treatment or being prescribed lower intensity treatment options (Byrne et al. 2025; Harrop et al. 2025; Muenzinger et al. 2025).

With increasing recognition of the co‐occurrence of EDs and higher weight, guidelines on the treatment of clinical obesity, published since 2020, have recommended assessment of ED symptoms (Ball et al. 2025; Hampl et al. 2023; National Institute for Health and Care Excellence 2026; Wharton et al. 2020). However, structured protocols on how to do this in practice have been lacking (Jebeile et al. 2026; McMaster et al. 2023). The first clinical practice guidelines for the management of EDs in people with higher weight recommend that all people presenting to health services seeking support with weight loss are screened for EDs (Ralph et al. 2022). The guideline recommends use of a brief ED screening questionnaire in the first instance, for example, the Eating Disorder Screen for Primary Care (Cotton et al. 2003). Individuals with a positive screen should undergo further ED assessment using a comprehensive assessment questionnaire, such as the Eating Disorder Examination Questionnaire (EDE‐Q) (Fairburn and Beglin 1994), and then progress to a clinical interview if required (Ralph et al. 2022). However, the authors note limitations of these instruments which have often been developed in people with lower weight and may contain language that could be stigmatizing for people with higher weight or items that may not be appropriate for this population (Ralph et al. 2022).

One challenge with identifying EDs in people with higher weight is that screening and assessment questionnaires have not been validated to identify the full spectrum of EDs (McMaster et al. 2023). In 2022, we published a systematic review including studies published to September 2020, examining the diagnostic accuracy of self‐report questionnaires against clinical interviews to identify EDs and disordered eating behaviors (DEBs) in adolescents and adults living with higher weight (House et al. 2022). The included studies had a strong focus on identifying binge eating and BED with few questionnaires in adults and none in adolescents evaluated to identify other EDs (e.g., BN, AAN). This is not surprising considering assessments for binge eating, such as the Binge Eating Scale (BES) (Gormally et al. 1982), were often developed with people with higher weight and/or in obesity treatment settings. The SCOFF was the only brief screening questionnaire for which diagnostic accuracy had been assessed to identify any ED in adults with higher weight, and sensitivity and specificity varied widely between studies (House et al. 2022). The SCOFF was designed to identify BN and anorexia nervosa (AN) according to the Diagnostic and Statistical Manual of Mental Disorders Fourth Edition (DSM‐IV) and may not be as useful for screening for the full spectrum of Diagnostic and Statistical Manual of Mental Disorders Fifth Edition (DSM‐5) EDs (Kutz et al. 2020). Similarly, the diagnostic accuracy of the EDE‐Q, one of the most used assessment questionnaires, had only been assessed to identify any ED in a single study and had slightly lower sensitivity in people with higher weight than those with lower weight (Mond et al. 2008). The EDE‐Q was originally developed to identify AN and BN in people with lower weight (Fairburn and Beglin 1994). Prior research has suggested that some items may perform differently in people with higher weight and that the factor structure may differ in this population, warranting further exploration (Costello et al. 2023; Grilo et al. 2013; Parker et al. 2016).

With continued calls for ED screening and assessment to be a routine part of care for people living with higher weight, particularly if engaging in obesity treatment, this review aimed to examine advances in the literature on the diagnostic accuracy of ED questionnaires for people with higher weight.

## 2 Methods

### 2.1 Protocol and Registration

The current review is an update of a review which examined the literature up to September 2020 (House et al. 2022). The methods were informed by the second version of the Cochrane Handbook for Systematic Reviews of Diagnostic Test Accuracy (Deeks et al. 2023) and the review has been reported according to the Preferred Reporting Items for Systematic Reviews and Meta‐Analyses (PRISMA) (Page et al. 2021), with reference to the extension for Diagnostic Test Accuracy studies where relevant (McInnes et al. 2018). The protocol for the updated review was registered with PROSPERO (https://www.crd.york.ac.uk/PROSPERO/view/CRD420251186115). The protocol was largely consistent with the original review. This is reported briefly, with changes from the original protocol outlined below (and shown in Table S1).

### 2.2 Eligibility Criteria

#### 2.2.1 Study Characteristics

**FIGURE 1:**
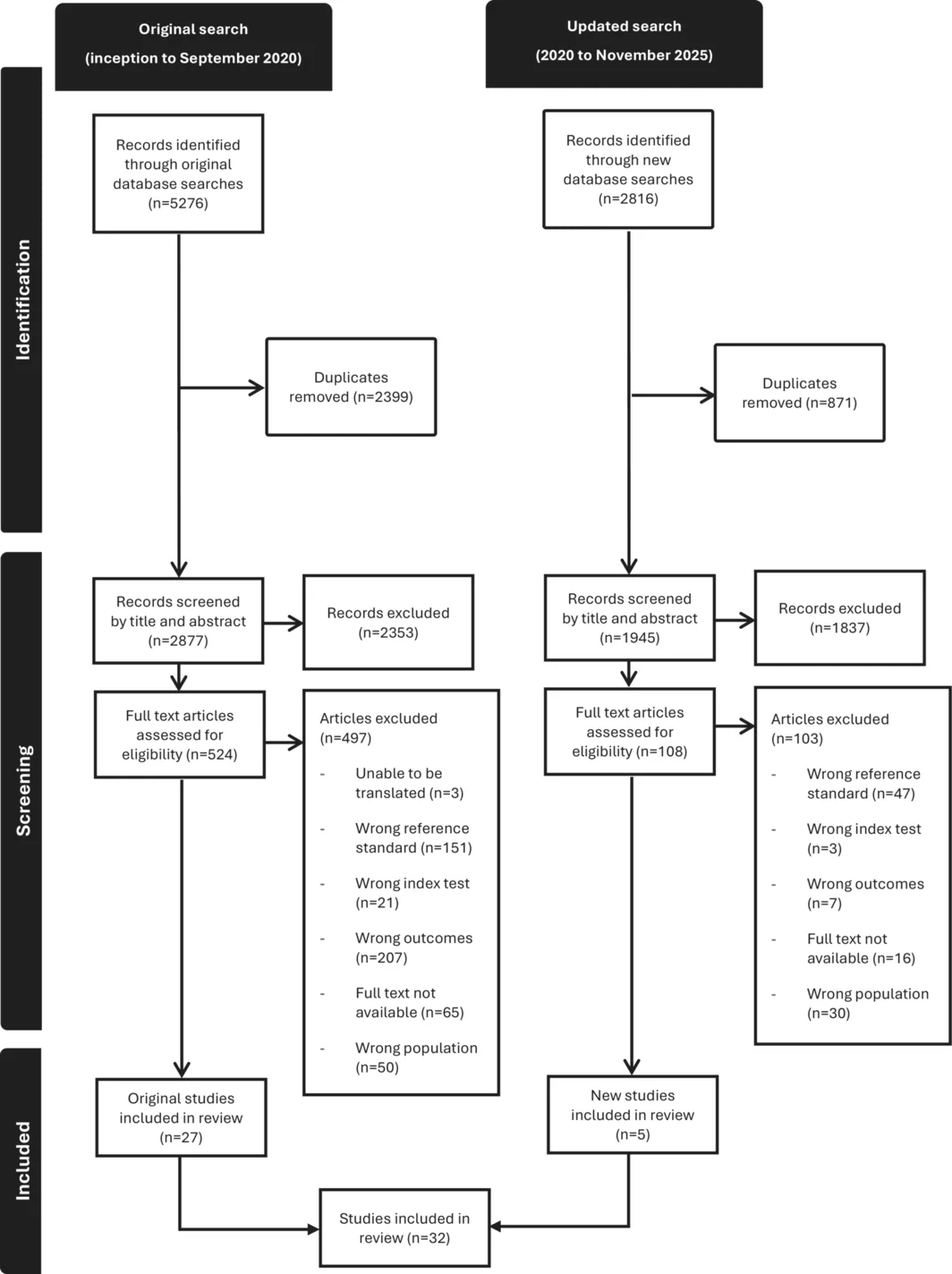
Preferred reporting items for systematic reviews and meta‐analysis (PRISMA) flow diagram of the literature search, screening, and study inclusion.

In the updated review, studies published in peer‐reviewed journals from 2020 to November 2025 were considered for inclusion. While studies published in a language other than English were excluded from the original review, in the updated review any studies identified in the original or updated search were translated using Google Translate to assess eligibility (this is reflected in the updated PRISMA diagram Figure 1).

#### 2.2.2 Study Design

Cross‐sectional studies conducted in any setting and reporting on the concurrent criterion validity of ED questionnaires, and reporting diagnostic accuracy outcomes (e.g., sensitivity, specificity, positive predictive value, negative predictive value) were included. Studies conducted during or following a clinical intervention were excluded to reduce risk of bias that may be introduced if treatment occurs between administration of the index test and reference standard (Deeks et al. 2023). Studies only assessing other components of validity (e.g., content validity, construct validity) were excluded. Literature reviews were excluded; reference lists of relevant reviews were checked for eligible studies.

#### 2.2.3 Participants

Included studies involved adolescents (10–17 years old) and adults (≥ 18 years old) with overweight or obesity, defined as:

– Adolescents: Adult equivalent body mass index (BMI) ≥ 25kg/m^2^, or BMI *z*‐score > 1, or BMI ≥ 85th percentile for sex and age, or clinical diagnosis of overweight or obesity
– Adults: BMI ≥ 25 kg/m^2^, or clinical diagnosis of overweight or obesity

If studies included participants across the weight spectrum and reported outcomes for participants with overweight/obesity separately, data for these participants were included. In the updated search, where identified studies reported that anthropometry was measured but diagnostic accuracy outcomes were not reported by BMI category, authors were contacted to request data for participants with overweight or obesity. Studies were included if these data were provided. Studies conducted in athletes, pregnant people or clinical populations (e.g., people living with diabetes, gastrointestinal illnesses, coeliac disease) were excluded as these populations may have unique factors influencing ED risk regardless of weight status that were beyond the scope of this review.

#### 2.2.4 Index Test

Included studies reported on the diagnostic accuracy of self‐report ED questionnaires, including both screening tools and assessment questionnaires. Studies regarding parent‐ or clinician‐reported questionnaires were excluded.

#### 2.2.5 Reference Standard

Studies assessing the diagnostic accuracy of self‐report questionnaires against a clinical diagnostic interview were included.

#### 2.2.6 Target Condition

Included studies assessed EDs based on the feeding and EDs listed in the DSM‐5 and DEBs that are symptoms of these EDs. Studies of questionnaires designed to assess other proposed EDs, which are not included in the DSM‐5, such as orthorexia nervosa or food addiction, were excluded.

### 2.3 Data Sources and Selection Process

Five electronic databases—CINAHL, EMBASE, MEDLINE, APA PsycINFO, and APA PsycTests—were systematically searched.

Search terms included Medical Subject Headings and keywords related to the population (adults and adolescents e.g., /Adolescent, Adult/, teen*.tw, adult*.tw), diagnosis (EDs and disordered eating, e.g., “Feeding and Eating Disorders”/, (disorder* adj4 eat*).tw, eating pathology.tw), index test (surveys and questionnaires, e.g., “Surveys and Questionnaires”/, questionnaire*.tw, survey*.tw), and outcomes (diagnostic accuracy, e.g., (Diagnos* adj3 accurac*).tw, sensitivity.tw, specificity.tw) of interest (see File S1 for the full search strategy).

Identified citations were imported into EndNote 20 (Clarivate Analytics) reference management software and duplicates removed. Remaining citations were uploaded into Covidence systematic review software (Veritas Health Innovation, Melbourne, Australia, available at www.covidence.org). Further duplicate removal, title and abstract and full‐text screening were conducted in Covidence. Screening was conducted in duplicate by independent reviewers (E.T.H., S.J.L., and I.R.J.), with conflicts resolved through discussion between reviewers. Exclusion reasons were recorded during full‐text screening.

### 2.4 Data Extraction

Data from newly identified studies were extracted using the Covidence customizable data extraction tool. Extraction was conducted by a single reviewer (S.J.L. or I.R.J. or E.T.H.) and cross‐checked by a second independent reviewer (E.T.H. or C.M.M. or S.J.L.). Extracted data included study details, participant characteristics, index test, reference standard, cut‐off points for the index test, and diagnostic accuracy outcomes.

### 2.5 Risk of Bias and Applicability

Risk of bias and applicability were assessed using the Quality Assessment of Diagnostic Accuracy Studies 2 (QUADAS‐2) tool (Whiting et al. 2011). The assessment was conducted in duplicate by independent reviewers (E.T.H., S.J.L., and I.R.J.), with conflicts resolved through discussion between the reviewers. Consistent with the recommendations for the use of the QUADAS‐2, a summary score was not generated for risk of bias; if a study was judged to be at “high” or “unclear” risk of bias in any of the four domains, it was deemed to be “at risk of bias”. If a study was judged to have “high” or “unclear” concerns regarding applicability in any of the three domains, it was deemed to have “concerns regarding applicability” (Whiting et al. 2011).

Risk of bias and applicability of the studies included in the original review (House et al. 2022) were re‐assessed using the QUADAS‐2 as the original review had used the earlier version of the tool (the QUADAS (Whiting et al. 2003)).

### 2.6 Data Synthesis

A narrative synthesis of data was conducted, guided by the Synthesis Without Meta‐Analysis (SWiM) guidelines (Campbell et al. 2020). We planned to give greatest weight to findings from studies with larger sample size and low risk of bias. When synthesizing and interpreting diagnostic accuracy outcomes to discuss implications for clinical practice, questionnaires with high sensitivity were prioritized over those with high specificity to reduce risk of false negatives. Sensitivity refers to the ability of a questionnaire to correctly identify people living with the target condition (i.e., an ED), it is the proportion of people with the condition who were identified by the test (i.e., true positives) out of all people with the condition (i.e., true positives and false negatives). Specificity refers to the ability of a questionnaire to correctly identify people who are not living with the target condition, it is the proportion of people without the condition who were correctly classified as not having the condition (i.e., true negatives) out of all those without the condition (i.e., true negatives and false positives) (Shreffler and Huecker 2025). The available evidence was visualized using evidence maps to illustrate the range of questionnaires that have had diagnostic accuracy assessed in adults and adolescents, the diagnoses which were examined, and the sensitivity of questionnaires in each study.

## 3 Results

Our updated search identified 2816 citations. No non‐English studies from the new and updated search were found to be eligible for the review. Enquiries were sent to authors of 23 studies to clarify the weight status of participants and/or request data separated by BMI category; authors of one study were able to provide required data for inclusion in the review (Altman et al. 2020). Five new studies were included in the review (Altman et al. 2020; Everett et al. 2021; Gormez et al. 2023; Jeong et al. 2023; Manasse et al. 2021), with a total of 32 studies included (Figure 1).

### 3.1 Included Studies

Twenty‐six included studies (including four new studies (Everett et al. 2021; Gormez et al. 2023; Jeong et al. 2023; Manasse et al. 2021)) were conducted in adults and six (including one new study (Altman et al. 2020)) were in adolescents.

Included studies were conducted in USA (*n* = 16), Italy (*n* = 3), Belgium (*n* = 2), Brazil (*n* = 2), the Netherlands (*n* = 2), Australia (*n* = 1), Austria (*n* = 1), France (*n* = 1), Sweden (*n* = 1), Switzerland (*n* = 1), Taiwan (*n* = 1), Türkiye (*n* = 1), and the UK (*n* = 1). Sample size ranged from 35 (Goldschmidt et al. 2007) to 1133 (Jeong et al. 2023). Mean age of adolescents ranged from 12.8 (Decaluwé and Braet 2004) to 14.3 (Goossens and Braet 2010) years and adults ranged from 27.6 (Mond et al. 2008) to 53.1 years (Calugi et al. 2020). Mean BMI (where reported for the sub‐sample of participants with higher weight) ranged from 35.2 (Orlandi et al. 2005) to 52.2 kg/m^2^ (Kalarchian et al. 2000) in adults. In adolescents BMI was reported in varying units between studies (e.g., adjusted BMI %, BMI percentile, or kg/m^2^). The proportion of females in included studies ranged from 49% (Liu et al. 2015) to 100% (Aardoom et al. 2012; Borges et al. 2005; De Zwaan et al. 1993; Freitas et al. 2006; Mond et al. 2008), with 12 studies having at least 80% females. Race or ethnicity of participants was reported in 15 studies, with 14 of 15 studies reporting more than 50% White participants, and 5 having at least 80%. Twenty‐one studies in adults and four in adolescents were conducted in clinical settings, including seven adult studies recruiting people presenting for bariatric surgery (Allison et al. 2008; de Man Lapidoth et al. 2007; Dymek‐Valentine et al. 2004; Grupski et al. 2013; Kalarchian et al. 2000; Parker et al. 2016; Quilliot et al. 2019). Characteristics of included studies are shown in Table S2.

### 3.2 Risk of Bias and Applicability

Risk of bias and applicability of included studies are shown in Table S3. All studies were deemed to be “at risk of bias.” Studies most often had “high” or “unclear” risk of bias in Domains 2 (index test) (*n* = 13 unclear, *n* = 9 high) and 4 (flow and timing) (*n* = 11 unclear, *n* = 18 high). None of the included studies had concerns regarding applicability. As all studies demonstrated risk of bias, weighting of results was primarily driven by sample size rather than study quality.

### 3.3 Diagnostic Accuracy Outcomes

To date, studies have examined the diagnostic accuracy of 13 questionnaires in adults and 5 in adolescents.

#### 3.3.1 Adults

**FIGURE 2:**
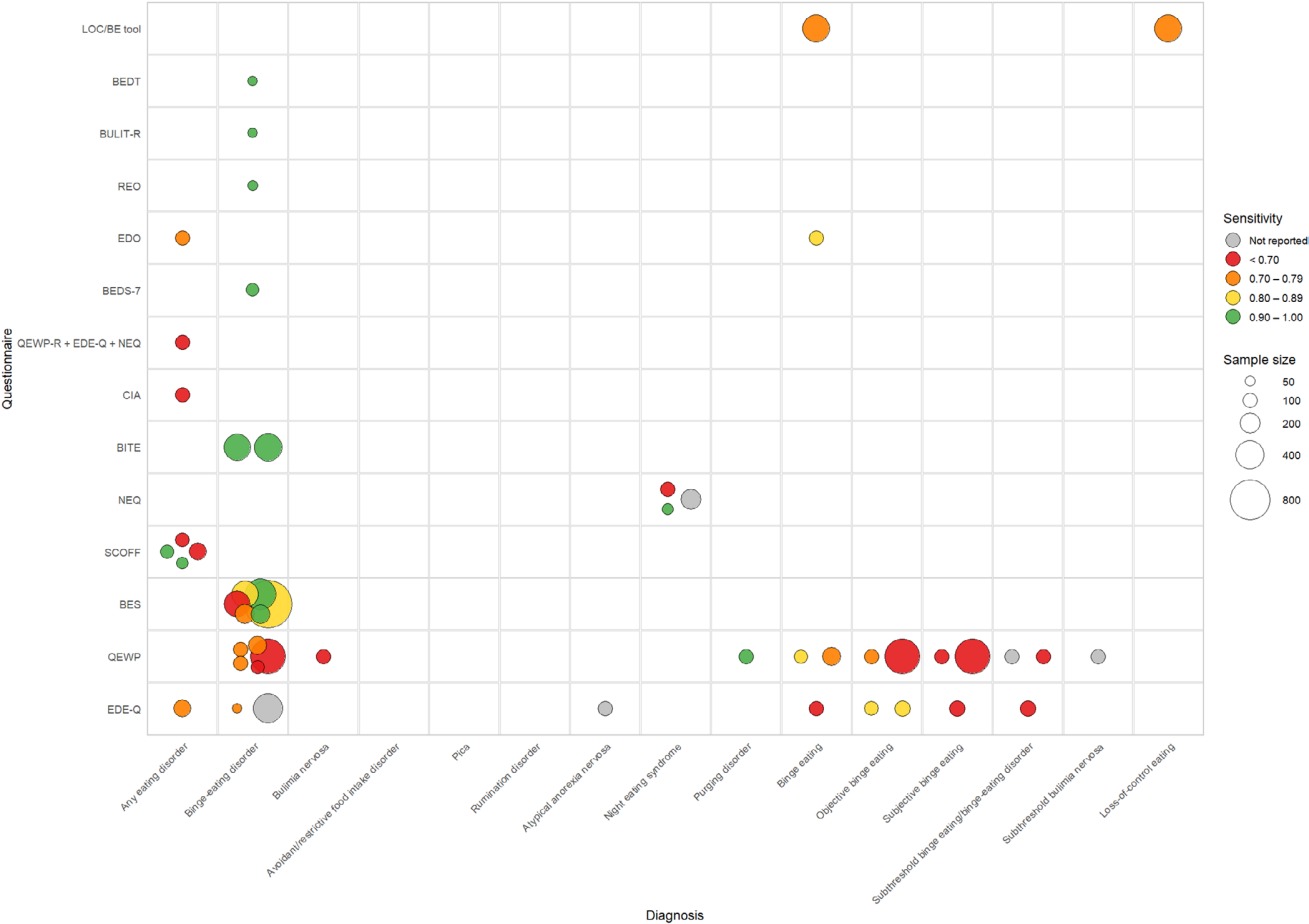
Evidence map showing the validation of eating disorder questionnaires in adults with higher weight. Each circle represents an individual study (some studies are represented more than once if they examined multiple questionnaires or diagnoses), the size of the circle represents the sample size, and the color represents the sensitivity of the questionnaire. Abbreviations: BE—binge eating, BEDS‐7—Binge‐Eating Disorder Screener‐7, BEDT—Binge‐Eating Disorder Test, BES—Binge Eating Scale, BITE—Bulimic Investigatory Test Edinburgh, BULIT‐R—Bulimia Test‐Revised, CIA—Clinical Impairment Assessment, EDE‐Q—Eating Disorder Examination Questionnaire, EDO—Eating Disorders in Obesity Questionnaire, LOC—loss of control, NEQ—Night Eating Questionnaire, QEWP(−R)—Questionnaire on Eating and Weight Patterns(‐Revised), REO—Risk Factors for Binge‐Eating in Overweight. (1) Solmi et al. (2015) reported sensitivity of the SCOFF for participants with overweight and obesity separately and combined, the combined sensitivity is shown. (2) The diagnostic accuracy of the SCOFF was assessed separately in males and females in one study (Liu et al. 2015), the findings are shown as separate dots, as sensitivity differed markedly between males (0.67) and females (0.96). (3) For the BES, studies used several different cut‐points, sensitivity is shown for the cut‐point that optimized sensitivity, this was: 15 in one study (Jeong et al. 2023), 17 in four studies (Freitas et al. 2006; Grupski et al. 2013; Quilliot et al. 2019; Ricca et al. 2000), 18 in one study (Gormez et al. 2023). (4) In the study by Jeong et al. (2023), diagnostic accuracy of the BES was assessed in a validation sample and then a replication sample. Sensitivity in the validation sample is displayed. (5) For the BITE, cut‐points of 10 and 20 (on the symptom sub‐scale and total score) were examined, sensitivity is displayed for a cut‐point of 10 (Orlandi et al. 2005; Ricca et al. 2000).

Figure 2 shows the range of questionnaires assessed in adults along with the diagnoses and behaviors for which diagnostic accuracy has been examined. The diagnostic accuracy of five questionnaires or combinations of questionnaires has been assessed to identify *any* ED, with only the SCOFF being examined in more than one study and demonstrating significant variability in sensitivity (0.67–1.0) between studies (Liu et al. 2015; Mond et al. 2008; Solmi et al. 2015). The diagnostic accuracy of eight questionnaires has been assessed to identify BED, with the BES being most frequently examined to identify BED and showing sensitivity greater than 0.8 in four out of six studies (at cut‐points of 15 to 17) (Freitas et al. 2006; Gormez et al. 2023; Grupski et al. 2013; Jeong et al. 2023; Quilliot et al. 2019; Ricca et al. 2000). The diagnostic accuracy of four questionnaires has been assessed to identify binge‐eating behaviors (including objective and subjective binge eating and subthreshold binge eating/BED). The Questionnaire on Eating Weight Patterns (QEWP) has been most frequently examined to identify binge‐eating behaviors (including subthreshold BED), having been evaluated in five studies showing variable sensitivity, with particularly low sensitivity to identify subjective binge eating (Borges et al. 2005; Calugi et al. 2020; Dymek‐Valentine et al. 2004; Hartmann et al. 2014; Parker et al. 2016). The diagnostic accuracy of single questionnaires has been assessed to identify BN and subthreshold BN (the Questionnaire on Eating and Weight Patterns Revised [QEWP‐R]), AAN (EDE‐Q), purging disorder (QEWP‐R), night eating syndrome (Night Eating Questionnaire [NEQ]), and loss‐of‐control (LOC) eating (LOC/Binge Eating Screening Tool), with only single studies conducted to assess diagnostic accuracy of questionnaires to identify BN/subthreshold BN, AAN, purging disorder, and LOC eating (Figure 2) (Allison et al. 2008; Hartmann et al. 2014; Manasse et al. 2021; Vander Wal et al. 2005). See Table 1 for definitions of the DEBs used in included studies.

**TABLE 1:** The disordered eating behaviors examined in included studies and the definitions of behaviors used in each study.

Disordered eating behavior	Number of studies	Definition(s) used
Objective binge eating/objective bulimic episodes (OBE)^a^	4 (Calugi et al. 2020; Everett et al. 2021; Goossens and Braet 2010; Parker et al. 2016)	In three of four studies, OBE was defined as eating an objectively large amount of food accompanied by a sense of loss of control (Everett et al. 2021; Goossens and Braet 2010; Parker et al. 2016). In one study, participants were classified as having OBE if episodes occurred at least once per week (Parker et al. 2016). In one study, OBE was measured but not defined (Calugi et al. 2020).
Binge eating	6 (Borges et al. 2005; de Man Lapidoth et al. 2007; Decaluwé and Braet 2004; Goldschmidt et al. 2007; Kalarchian et al. 2000; Manasse et al. 2021)	In five studies, the definition of ‘binge eating’ was consistent with that of OBE, that is, eating an objectively large amount of food with a sense of loss of control (de Man Lapidoth et al. 2007; Decaluwé and Braet 2004; Goldschmidt et al. 2007; Kalarchian et al. 2000; Manasse et al. 2021). These studies employed differing frequency criteria for categorizing participants as engaging in binge eating: – 2 binges per week on average (Kalarchian et al. 2000) – Any binge eating in the past 3 months (de Man Lapidoth et al. 2007) – ≥ 12 episodes over the past 3 months (Manasse et al. 2021) – Any binge eating in the past 28 days (Decaluwé and Braet 2004; Goldschmidt et al. 2007) In contrast, one study categorized participants as experiencing ‘binge eating in general’ (Borges et al. 2005). This included participants with BED, ‘binge‐eating syndrome’ (used to describe those with recurrent episodes of overeating and loss of control + three associated symptoms of loss of control) with or without distress, ‘binge eating’ (recurrent episodes of overeating with loss of control), bulimia nervosa (recurrent episodes of overeating with loss of control + three associated symptoms of loss of control + undue influence of weight and shape on self‐evaluation + frequent compensatory behavior for weight control).
LOC eating	2 (Altman et al. 2020; Manasse et al. 2021)	LOC eating refers to the subjective feeling of loss of control while eating. In both studies examining LOC eating, participants were classified as experiencing LOC eating, if they reported any LOC eating within the past 3 months (Altman et al. 2020; Manasse et al. 2021).
Objective overeating	1 (Goossens and Braet 2010)	Eating an objectively large amount of food without experiencing a sense of loss of control over eating (Goossens and Braet 2010).
Subjective binge eating/subjective bulimic episodes (SBE)^a^	3 (Calugi et al. 2020; Goossens and Braet 2010; Parker et al. 2016)	In one study, SBE was defined as experiencing a sense of loss of control while eating an amount of food that would not be viewed as unusually large by others (Parker et al. 2016). Another study, added to this definition, that the subject considers the amount of food as large though others would not (Goossens and Braet 2010). One study did not provide a definition of SBE (Calugi et al. 2020).
Self‐induced vomiting	1 (Goossens and Braet 2010)	Not further described in the included study
Laxative/diuretic misuse	1 (Goossens and Braet 2010)	Not further described in the included study
Appetite depressant misuse	1 (Goossens and Braet 2010)	Not further described in the included study
Excessive exercise	1 (Goossens and Braet 2010)	Not further described in the included study
Subthreshold binge eating	1 (Parker et al. 2016)	Not further described in the included study

Abbreviations: BED—binge‐eating disorder, LOC—loss of control, OBE—objective binge eating/objective bulimic episode, SBE—subjective binge eating/subjective bulimic episode.

^a^ The terms objective/subjective 'binge eating' or 'bulimic episode' are used in different studies but describe the same phenomenon. The term objective or subjective 'binge eating' is used in this review.

Newly included studies examined the diagnostic accuracy of three different questionnaires in adults (the EDE‐Q, BES, and LOC/Binge Eating Screening Tool); the results of newly included studies are discussed in more detail. These findings are synthesized by index test with the new evidence compared to the literature included in the 2022 review (House et al. 2022) (Table 2).

**TABLE 2:** A summary of the diagnostic accuracy of eating disorder questionnaires in adults, new or updated findings that have arisen from the update to the review are shown in bold.

Adults				
Questionnaire	Number of studies, sample size (range of *n*)	Diagnoses	Sensitivity/specificity by diagnosis	Considerations
Eating Disorder Examination Questionnaire (EDE‐Q)	**7 studies** (Aardoom et al. 2012; **Everett et al.** 2021; Hartmann et al. 2014; Kalarchian et al. 2000; Mond et al. 2008; Parker et al. 2016; Vander Wal et al. 2011), *n* = 41–433	ED, BED, AAN, BE^a^	ED (cut‐point 3.1)—0.77/0.73 BED—0.40–0.87/0.62–1.0 AAN—sensitivity and specificity NR **BE—0.16‐0.88/0.54–0.89**	Sensitivity to identify OBE was greater than SBE (Everett et al. 2021; Parker et al. 2016) The restraint subscale had lower sensitivity to identify BED than other subscales or the global score (Vander Wal et al. 2011) There is evidence that the original 4‐factor structure may have poor fit and psychometric properties when used in people with higher weight (Costello et al. 2023; Grilo et al. 2013) Only one study reported a cut‐point for identifying any ED, using a cut‐point of 3.1 (Mond et al. 2008). Another study (not included in this review) identified a cut‐point of 3.15 for people with overweight (i.e., BMI 25–29.9 kg/m^2^) and 3.26 for people with obesity (i.e., BMI ≥ 30 kg/m^2^) (Rø et al. 2015). These cut‐points have been developed in females.
Binge Eating Scale (BES)	**6 studies** (Freitas et al. 2006; **Gormez et al.** 2023; Grupski et al. 2013; **Jeong et al.** 2023; Quilliot et al. 2019; Ricca et al. 2000), ***n* = 178–1133**	BED	**Cut‐point of 15–0.72–0.82/0.67–0.69** **Cut‐point of 17–0.51‐0.98/0.48–0.77** **Cut‐point of 18–0.72/0.79** Cut‐point of 27–0.37‐0.61/0.95–0.96	The BES was developed in adults seeking obesity treatment (Gormally et al. 1982) A lower cut‐point (15–18) is likely to be more suitable for screening purposes to reduce the risk of false negatives (Freitas et al. 2006; Gormez et al. 2023; Grupski et al. 2013; Jeong et al. 2023; Ricca et al. 2000)
Questionnaire on Eating and Weight Patterns (QEWP/QEWP‐revised)	6 studies (Borges et al. 2005; Calugi et al. 2020; De Zwaan et al. 1993; Dymek‐Valentine et al. 2004; Hartmann et al. 2014; Parker et al. 2016), *n* = 89–604	BED, BN, Purging disorder, BE^a^/subthreshold BED, subthreshold BN	BED—0.49‐0.73/0.80–0.93^b^ BN—0.5/0.0 Purging disorder—1.0/0.01 BE/subthreshold BED—0.07‐0.88/0.63–1.0 Subthreshold BN—sensitivity and specificity NR	Direct comparison between the QEWP‐R and EDE‐Q in adults seeking bariatric surgery suggests the EDE‐Q may better identify patients with binge eating who are experiencing body weight and shape concerns (Elder et al. 2006)
SCOFF	3 studies (Liu et al. 2015; Mond et al. 2008; Solmi et al. 2015), *n* = 63–178	ED	Cut‐point of 2–0.69–1.0/0.59–0.83 Cut‐point of 3–0.67/0.65	Sensitivity was higher in people with overweight than obesity in one study (Solmi et al. 2015) One study indicates a cut‐point of 3 may be more appropriate for males than the usual cut‐point of 2 (Liu et al. 2015) Several short screening tools have been developed since the SCOFF, including the Screen for Disordered Eating (SDE) (Maguen et al. 2018), Eating Disorder Screen for Primary Care (ESP) (Cotton et al. 2003), and InsideOut Institute Screener (IOI‐Screener) (Bryant et al. 2021), none have been validated in people with higher weight. However, they have demonstrated greater sensitivity than the SCOFF in community samples (Bryant et al. 2021; Maguen et al. 2018)
Night Eating Questionnaire (NEQ)	3 studies (Allison et al. 2008; Hartmann et al. 2014; Vander Wal et al. 2005), *n* = 59–194	NES	< 0.01 (cut‐point of 30)–1.0 (cut‐points of 5–10)/0.0–0.4 (cut‐points of 5–10)	The 9‐item version used in the Weight and Lifestyle Inventory has high sensitivity at cut‐points of 5–10 (depending on the definition of NES used), however, specificity is low (Vander Wal et al. 2005) A single question regarding night eating, “To what extent does snacking after dinner contribute to your weight problem?”, rated on a scale of 1–5 had sensitivity of ≥ 0.8 at a cut‐point of 4 (Vander Wal et al. 2005) The diagnostic accuracy of the 14‐item version has shown variability, it had high NPV in one study (Allison et al. 2008) but low sensitivity in another study at the recommended cut‐point of 30 (Hartmann et al. 2014)
Bulimic Investigatory Test, Edinburgh (BITE)	2 studies (Orlandi et al. 2005; Ricca et al. 2000), *n* = 344–388	BED	Cut‐point of 10 (total score‐symptom sub‐scale)—0.91–0.93/0.51–0.55 Cut‐point of 20 (total score‐symptom sub‐scale) —0.33‐0.41/0.92	A cut‐point of 10 (using either the total score or symptom sub‐scale) is likely to be more suitable than 20 for screening purposes to avoid the risk of false negatives (Orlandi et al. 2005; Ricca et al. 2000) The BITE can also be used to assess risk of bulimia nervosa (Henderson and Freeman 1987), however, validation in people with higher weight has not occurred
Clinical Impairment Assessment (CIA)	1 study (Hartmann et al. 2014), *n* = 100	ED	Cut‐point of 16–0.59/0.74^c^	The CIA differs from other included questionnaires as it focusses on assessing impairment related to eating disorder psychopathology across domains of psychosocial functioning (Vannucci et al. 2012) May not be a useful screener for DSM‐5 eating disorders in people with higher weight due to high number of false positives and false negatives (Hartmann et al. 2014)
Binge‐Eating Disorder Screener‐7 (BEDS‐7)	1 study (Herman et al. 2016), *n* = 80	BED	1.0/0.34–0.48 (dependent on BMI range)	The BEDS‐7 demonstrated 100% sensitivity and may act as a useful brief screener, though it may result in a high number of false positives (Herman et al. 2016)
Eating Disorders in Obesity questionnaire (EDO)	1 study (de Man Lapidoth et al. 2007), *n* = 97	ED, BE	ED—0.75/0.99 BE—0.82/0.83	The EDO was designed to assess the spectrum of EDs involving binge‐eating behaviors (with or without purging), it may not be able to identify people with restrictive eating behaviors without binge eating (de Man Lapidoth et al. 2007) The diagnostic accuracy of the EDO has not been further assessed since it's development
Risk Factors for Binge‐Eating Disorder in Overweight (REO)	1 study (Wever et al. 2018), *n* = 50	BED	Cut‐point of 83.5–0.95/0.82	The REO showed good diagnostic accuracy to identify BED in development (Wever et al. 2018), however, findings are yet to be replicated in further studies
Bulimia Test—Revised (BULIT‐R)	1 study (Vander Wal et al. 2011), *n* = 41	BED	Cut‐point of 80–1.0/0.96	The BEDT was derived from the BULIT‐R (Thelen et al. 1991; Vander Wal et al. 2011) The BEDT may be preferred if only screening for BED due to its brevity (23‐items) compared to the BULIT‐R (36‐items) (Thelen et al. 1991; Vander Wal et al. 2011) The BULIT‐R may be useful for identifying compensatory behaviors not captured in the BEDT (Vander Wal et al. 2011) Diagnostic accuracy findings have not been replicated in studies using DSM‐5 criteria
The Binge‐Eating Disorder Test (BEDT)	1 study (Vander Wal et al. 2011), *n* = 41	BED	Cut‐point of 75–1.0/1.0	
**LOC/binge eating screening tool**	**1 study (Manasse et al.** 2021 **), *n* = 364**	**BE, LOC**	**BE—0.64‐0.85/0.70–0.82** **LOC—0.72–0.79/0.58–0.79**	Authors suggest diagnostic accuracy may be improved through further explanation of LOC and BE using metaphors or examples, as well as addition of items regarding drivers and contexts in which behaviors may occur (Manasse et al. 2021) Diagnostic accuracy findings are yet to be replicated in subsequent studies
Combination of QEWP‐R + EDE‐Q + NEQ	1 study (Hartmann et al. 2014), *n* = 100	ED	0.47/0.78	Authors assessing this combination of questionnaires proposed several modifications to seek to improve diagnostic accuracy (Hartmann et al. 2014): ○ **QEWP‐R:** adapt duration and scoring to reflect DSM‐5 BN and BED criteria (this has been addressed in the QEWP‐5 (Yanovski et al. 2015)) and to identify limited duration and frequency of behaviors indicative of subthreshold disorders ○ **EDE‐Q:** add assessment of significant weight loss to detect AAN ○ **NEQ:** include assessment of daytime binge eating to differentiate BED, BN, and NES

Abbreviations: AAN—atypical anorexia nervosa, BE—binge eating, BED—binge‐eating disorder, BEDS‐7—Binge‐Eating Disorder Screener‐7, BEDT—Binge‐Eating Disorder Test, BES—Binge Eating Scale, BITE—Bulimic Investigatory Test Edinburgh, BMI—body mass index, BULIT‐R—Bulimia Test‐Revised, CIA—Clinical Impairment Assessment, DSM‐5—Diagnostic and Statistical Manual of Mental Disorders Fifth Edition, EDE‐Q—Eating Disorder Examination Questionnaire, EDO—Eating Disorders in Obesity questionnaire, LOC—loss of control, NEQ—Night Eating Questionnaire, NES—Night Eating Syndrome, NR—not reported, OBE—objective binge eating, NPV—negative predictive value, QEWP—Questionnaire on Eating and Weight Patterns, QEWP‐R—Questionnaire on Eating and Weight Patterns‐Revised, REO—Risk Factors for Binge‐Eating in Overweight, SBE—subjective binge eating.

^a^ These range of sensitivity and specificity reported for identifying BE includes reported diagnostic accuracy to identify objective binge eating, subjective binge eating and binge eating in general.

^b^ The upper range of specificity observed reflects the highest specificity reported using DSM‐5 criteria, Hartmann et al. (2014) found the QEWP‐R to have higher specificity (0.96) when DSM‐IV criteria were used to diagnose BED.

^c^ The sensitivity and specificity of the CIA was assessed using DSM‐IV and DSM‐5 criteria, diagnostic accuracy using DSM‐5 criteria is shown.

##### 3.3.1.1 EDE‐Q

Seven studies have examined the diagnostic accuracy of the EDE‐Q in adults with higher weight, with one study validating the EDE‐Q to identify any DSM‐IV ED (Mond et al. 2008), one to identify AAN (Hartmann et al. 2014), two to identify BED (Aardoom et al. 2012; Vander Wal et al. 2011), and three to identify binge eating (Everett et al. 2021; Kalarchian et al. 2000; Parker et al. 2016).

One recent study (Everett et al. 2021), conducted in the USA with 94 participants during a baseline assessment for a behavioral weight loss trial, assessed the diagnostic accuracy of the EDE‐Q against the Eating Disorder Examination (EDE) interview for the identification of objective binge eating. The results indicated that the EDE‐Q had higher sensitivity (0.80) than specificity (0.54) (Everett et al. 2021). The observed sensitivity was within the range identified in the original review when the EDE‐Q was used to identify binge eating in 2 studies (Kalarchian et al. 2000; Parker et al. 2016) with sample sizes of 98 and 117; however, specificity was lower than previously observed (previously specificity ranged from 0.62 to 0.89) (Kalarchian et al. 2000; Parker et al. 2016).

##### 3.3.1.2 BES

Six studies in total have examined the diagnostic accuracy of the BES in adults with higher weight, with all studies validating the BES to identify BED. Two newly included studies assessed the diagnostic accuracy of the BES to identify BED among patients in clinical obesity treatment settings (Gormez et al. 2023; Jeong et al. 2023). The larger of the 2 studies was conducted in USA with 1133 adults presenting for metabolic and bariatric surgery and assessed the diagnostic accuracy of the BES against a semi‐structured clinical interview using DSM‐5 criteria for BED (Jeong et al. 2023). In this study, they identified a cut‐point of 15 as offering the best balance between sensitivity and specificity. At this cut‐point the BES had higher sensitivity (0.72–0.82) than specificity (0.67–0.69). While at a cut‐point of 17 (usually used to define “mild to moderate” binge eating), the BES had lower sensitivity (0.58–0.75) than specificity (0.75–0.77) (Jeong et al. 2023). The proportion of correct predictions in the overall sample (referred to by the authors as the “hit rate”) was slightly higher at a cut‐off of 17 (0.72–0.75) than 15 (0.70–0.71). Overall, they found the BES to have an Area Under the Curve (AUC) of 0.76–0.80, which authors interpreted as acceptable diagnostic performance. The smaller study (Gormez et al. 2023) examined the Turkish version of the BES against the Structured Clinical Interview for DSM‐5‐Disorders—Clinician Version (SCID‐5‐CV) among 188 adults presenting to an outpatient obesity clinic. This study used a cut‐point of 18, finding the BES to have moderate sensitivity (0.72) and specificity (0.79). The AUC was similar to that observed in the larger study at 0.79.

Compared to the 4 studies (sample size *n* = 178–473) identified in the original review (Freitas et al. 2006; Grupski et al. 2013; Quilliot et al. 2019; Ricca et al. 2000) these studies provided new evidence regarding the diagnostic accuracy of the BES when using cut‐points of 15 or 18, as well as the AUC, which had not been previously reported. The sensitivity and specificity at a cut‐point of 17 were similar to the range observed in earlier studies (sensitivity—0.51–0.98, specificity—0.48–0.76) (Freitas et al. 2006; Grupski et al. 2013; Quilliot et al. 2019; Ricca et al. 2000).

##### 3.3.1.3 Loss of Control (LOC)/Binge Eating Screening Tool

One newly identified study reported on the development and validation of a new screening tool to identify LOC eating and full‐threshold binge eating (Manasse et al. 2021). The study included 364 adults presenting to a clinical trial center for trials related to eating and weight disorders. The diagnostic accuracy of the LOC/binge eating screening tool was assessed against the EDE. It was found to have moderate sensitivity and specificity to identify LOC eating (0.78/0.77) and full‐threshold binge eating (0.75/0.74) (Manasse et al. 2021). Overall, the derived discriminant function was significant for the identification of both LOC eating and full‐threshold binge eating, suggesting the tool could discriminate between cases and non‐cases (Manasse et al. 2021).

#### 3.3.2 Adolescents

**FIGURE 3:**
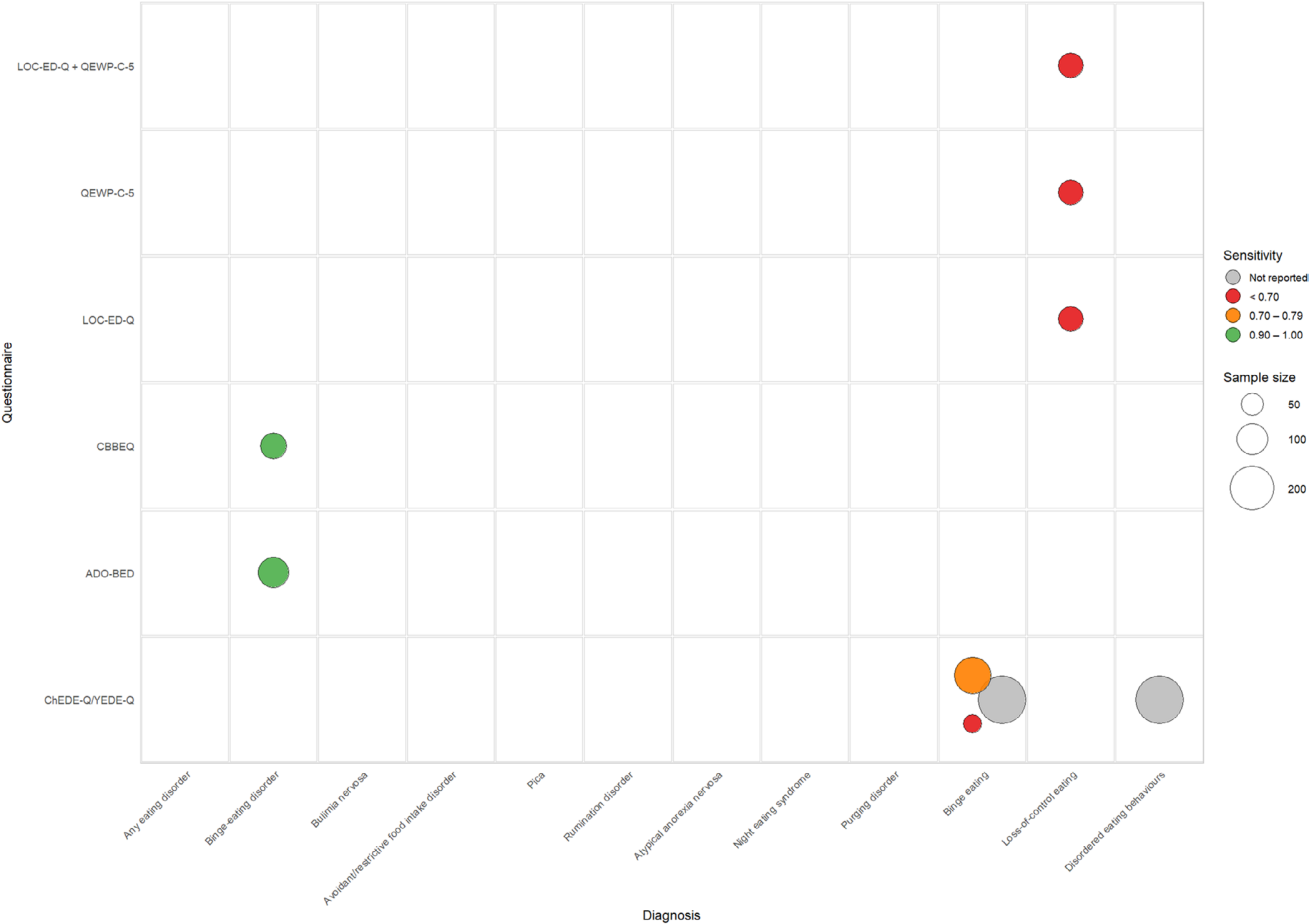
Evidence map showing the validation of eating disorder questionnaires in adolescents with higher weight. Each circle represents an individual study, the size of the circle represents the sample size, and the color represents the sensitivity of the questionnaire. Abbreviations: ADO‐BED—Adolescent Binge‐Eating Disorder Questionnaire, CBBEQ—Children's Brief Binge Eating Questionnaire, ChEDE‐Q—Children's Eating Disorder Examination Questionnaire, LOC‐ED‐Q—Loss of Control Eating Disorder Questionnaire, QEWP‐C‐5—Questionnaire on Eating and Weight Patterns‐5 Children/Adolescent, YEDE‐Q—Youth Eating Disorder Examination Questionnaire. (1) The ADO‐BED sensitivity depicted is for the use of the first two questions as a screener, where a “yes” on either question is considered a positive screen (Chamay‐Weber et al. 2017).

Figure 3 shows the range of questionnaires for which diagnostic accuracy has been assessed in adolescents and the diagnoses and behaviors questionnaires have been validated to identify. In adolescents, the diagnostic accuracy of two questionnaires (the Children's Brief Binge Eating Questionnaire [CBBEQ] and Adolescent Binge‐Eating Disorder Questionnaire [ADO‐BED]) has been assessed to identify BED in single studies, with sensitivity of 1.0 (Chamay‐Weber et al. 2017; Franklin et al. 2019). The diagnostic accuracy of child and youth versions of the EDE‐Q has been assessed in three studies to identify binge‐eating behaviors, showing low to moderate sensitivity (0.57–0.79) (Decaluwé and Braet 2004; Goldschmidt et al. 2007; Goossens and Braet 2010), and in one study to identify other DEBs (Goossens and Braet 2010). The diagnostic accuracy of two questionnaires (Questionnaire on Eating and Weight Patterns‐5 Children/Adolescent [QEWP‐C‐5] and Loss‐of‐Control Eating Disorder Questionnaire [LOC‐ED‐Q]) has been examined to identify LOC eating (Table 3).

**TABLE 3:** A summary of the diagnostic accuracy of eating disorder questionnaires in adolescents, new or updated findings that have arisen from the update to the review are shown in bold.

Adolescents				
Questionnaire	Number of studies, sample size (range of *n*)	Diagnoses	Sensitivity/specificity by diagnosis	Considerations
Children's/Youth Eating Disorder Examination Questionnaire (ChEDE‐Q/YEDE‐Q)	3 studies (Decaluwé and Braet 2004; Goldschmidt et al. 2007; Goossens and Braet 2010), *n* = 35–235	BE, DEBs	BE—0.57‐0.79/0.68–1.0 DEBs—sensitivity and specificity NR	The ChEDE‐Q/YEDE‐Q consistently result in higher subscale scores than the ChEDE interview (Decaluwé and Braet 2004; Goldschmidt et al. 2007; Goossens and Braet 2010) The optimal cut‐point has not been specified in any included study
Adolescent Binge‐Eating Disorder Questionnaire (ADO‐BED)	1 study (Chamay‐Weber et al. 2017), *n* = 94	BED	1.0/0.27 (if using first two questions for screening)	The first question regarding eating in the absence of hunger was particularly sensitive for identifying BED risk (Chamay‐Weber et al. 2017) Diagnostic accuracy findings are yet to be replicated in subsequent studies
Children's Brief Binge Eating Questionnaire (CBBEQ)	1 study (Franklin et al. 2019), *n* = 70	BED	Cut‐point 8–1.0/0.93	Responses to items related to non‐hungry eating (Q1), loss of control (Q2) and emotional eating (Q4) closely aligned with diagnostic interviews (Franklin et al. 2019) Diagnostic accuracy findings are yet to be replicated in subsequent studies
**Loss of Control Eating Disorder Questionnaire (LOC‐ED‐Q)**	**1 study (Altman et al.** 2020 **), *n* = 65**	**LOC**	**0.42/0.91**	When used independently or in combination, lack sensitivity to be useful for screening purposes (Altman et al. 2020) Authors suggest research is needed to identify the best way to improve understanding of LOC as a construct in questionnaires (Altman et al. 2020)
**Questionnaire on Eating and Weight Patterns‐5 Children/Adolescent (QEWP‐C‐5)**	**1 study (Altman et al.** 2020 **), *n* = 65**	**LOC**	**0.42/0.93**	
**Combination of LOC‐ED‐Q and QEWP‐C‐5**	**1 study (Altman et al.** 2020 **), *n* = 65**	**LOC**	**0.50/0.87**	

Abbreviations: ADO‐BED—Adolescent Binge‐Eating Disorder Questionnaire, BE—binge eating, BED—binge‐eating disorder, CBBEQ—Children's Brief Binge Eating Questionnaire, ChEDE—Children's Eating Disorder Examination, ChEDE‐Q—Children's Eating Disorder Examination Questionnaire, DEB—disordered eating behavior, LOC—loss of control, LOC‐ED‐Q—Loss of Control Eating Disorder Questionnaire, NR—not reported, QEWP‐C‐5—Questionnaire on Eating and Weight Patterns‐5 Children/Adolescent, YEDE‐Q—Youth Eating Disorder Examination Questionnaire.

One newly included study examined the diagnostic accuracy of two questionnaires in adolescents (the QEWP‐C‐5 and LOC‐ED‐Q). The results of this study are described in more detail.

##### 3.3.2.1 Questionnaire on Eating and Weight Patterns‐5 Children/Adolescent (QEWP‐C‐5) and Loss‐Of‐Control Eating Disorder Questionnaire (LOC‐ED‐Q)

The only newly identified study conducted in adolescents examined the diagnostic accuracy of both the QEWP‐C‐5 and LOC‐ED‐Q against the EDE (or child version) to identify LOC eating (Altman et al. 2020). The study included children and adolescents enrolled in a non‐interventional longitudinal study, including a sub‐sample of 65 adolescents (aged 10–17 years) with BMI *z*‐score > 1. In this sub‐group both questionnaires demonstrated low sensitivity (both 0.42) and high specificity (LOC‐ED‐Q: 0.91, QEWP‐C‐5: 0.93), when used in combination sensitivity increased to 0.5 and specificity reduced to 0.87 (Altman et al. 2020). The overall “efficiency” of the questionnaires was also examined (i.e., the proportion of true positives and true negatives out of all participants) and was similar for both questionnaires individually (LOC‐ED‐Q: 0.82, QEWP‐C‐5: 0.83) and when used in combination (0.80).

## 4 Discussion

In this systematic review of the diagnostic accuracy of self‐report questionnaires to identify EDs and DEBs in people with higher weight, we have identified 32 studies, with 5 published in the last 5 years. Of concern, there has been only one new study conducted in adolescents with higher weight. All newly identified studies assessed BED, binge‐eating behaviors and/or LOC eating, with no advancement in assessment of other EDs or with assessments considering the full spectrum of ED presentations in people with higher weight within one questionnaire. Newly identified studies build on prior literature on the diagnostic accuracy of the EDE‐Q and BES for the assessment of binge‐eating behaviors and BED, respectively. It is promising that the diagnostic accuracy of one new questionnaire in adults and two in adolescents has been assessed to identify LOC eating. The sensitivity and specificity of the tool used in adults were within the range identified for the EDE‐Q and QEWP when used to identify binge eating; however, another tailored tool such as the Eating Disorders in Obesity (EDO) questionnaire (de Man Lapidoth et al. 2007) may be preferred given similar diagnostic accuracy and its ability to assess a broader range of EDs. In adolescents, neither of the newly assessed questionnaires appears to have sufficient sensitivity to be used to screen for LOC eating.

Most of the literature regarding the diagnostic accuracy of ED questionnaires for people with higher weight remains focused on the identification of binge‐eating behaviors and BED, with only five studies (reporting on four different questionnaires and one combination of questionnaires) evaluating questionnaires to identify *any* ED (de Man Lapidoth et al. 2007; Hartmann et al. 2014; Liu et al. 2015; Mond et al. 2008; Solmi et al. 2015). There is a lack of sufficiently sensitive questionnaires to identify the full spectrum of EDs in people with higher weight, with few tools to identify restrictive EDs/behaviors in adults and none in adolescents. People with higher weight who present with restrictive EDs are at risk of being overlooked due to misperceptions regarding the characteristics of patients with such disorders, particularly a lack of understanding that these disorders can occur across the weight spectrum (Harrop et al. 2025). This is despite adolescents with a history of higher weight presenting for treatment of restrictive EDs (AN and AAN) with greater percentage weight loss and comparable medical complications and psychiatric comorbidities to people without a history of higher weight (Brennan et al. 2023; Kennedy et al. 2017; Lebow et al. 2015; Matthews et al. 2023). In addition, we have not identified any questionnaires with established diagnostic accuracy to identify Avoidant/Restrictive Food Intake Disorder (ARFID) in people with higher weight. This is particularly pertinent as there is emerging evidence that individuals with comorbid ARFID and higher weight may present with different diagnostic characteristics than people with lower weight and thus may not be well recognized (Merz et al. 2025). Overall, there remains a significant gap in the literature, making it difficult to recommend any single questionnaire for identifying the full spectrum of EDs in adults and adolescents with higher weight.

### 4.1 Brief Screening Questionnaires

In the past 5 years, there has been no progress in the assessment of the diagnostic accuracy of brief ED screening tools. The SCOFF remains the only such tool for which diagnostic accuracy has been examined to identify *any* ED in people with higher weight. The BEDS‐7 remains the only brief screening tool with established diagnostic accuracy to identify BED in adults (Herman et al. 2016), and the CBBEQ (Franklin et al. 2019) and first two questions of the ADO‐BED (Chamay‐Weber et al. 2017) may be useful brief screening options for BED in adolescents. However, the promising diagnostic accuracy findings in the initial validation of these tools have not been replicated in further research in the past 5 years.

There are several brief screening tools that have been developed for use in primary care, to identify EDs beyond AN and BN, which have been shown to have greater sensitivity than the SCOFF in adults, including the Eating Disorder Screen for Primary Care (Cotton et al. 2003), Screen for Disordered Eating (Maguen et al. 2018), and InsideOutInstitute Screener (Bryant et al. 2021). While these tools were developed in community samples which may include people with higher weight, their diagnostic accuracy has not been examined separately for people with higher weight. Promisingly, there is research underway to develop a screening questionnaire to identify EDs in children and adolescents across the weight spectrum—the “Screening Kids' And Teens' Eating” questionnaire (Button et al. 2025). Assessing the diagnostic accuracy of brief screening questionnaires in people with higher weight should be a research priority. These tools are essential in resource‐limited settings to efficiently identify people with likely EDs who require further assessment, supporting implementation of clinical practice guidelines in routine care (Ralph et al. 2022).

### 4.2 Comprehensive Assessment Questionnaires

There has also been no progress in research exploring the diagnostic accuracy of comprehensive assessment questionnaires designed to identify all ED diagnoses. To date, there has still been only one study examining the diagnostic accuracy of the EDE‐Q to identify any ED (Mond et al. 2008). This study recruited young adult women and used DSM‐IV criteria to diagnose EDs (Mond et al. 2008), finding that a higher EDE‐Q cut‐off score was required in people with higher weight than those with BMI < 27.5 kg/m^2^. This is consistent with other research, not eligible for this review, which has also identified higher optimal cut‐off scores for the EDE‐Q in people with higher weight (Rø et al. 2015). In contrast, newly identified studies build on prior literature on the validation of the EDE‐Q and BES for the assessment of binge‐eating behaviors and BED, respectively. The BES may be a useful assessment questionnaire for BED, given it has shown sensitivity > 0.80 in four out of six included studies. In contrast, the utility of the EDE‐Q to assess binge‐eating behaviors is unclear, with significant variability in findings between studies. Overall, there remains a significant gap in the literature to inform the selection of appropriate ED assessment questionnaires to identify EDs other than BED in people with higher weight.

### 4.3 Diagnostic Accuracy for Specific Populations

In our original 2020 search, we identified diagnostic accuracy studies of three questionnaires to identify binge eating, other DEBs, and BED in adolescents living with higher weight. At the time, the diagnostic accuracy of questionnaires to identify other ED diagnoses had not been assessed (House et al. 2022). As of 2025, we have identified one additional study examining the diagnostic accuracy of two questionnaires to identify LOC eating in adolescents. However, there remains a significant gap in the literature, with these new questionnaires demonstrating low sensitivity and with no studies identified that examine the diagnostic accuracy of questionnaires to identify non‐binge EDs in adolescents with higher weight. Adolescence and young adulthood are the peak period for the onset of EDs (Favaro et al. 2019), with the peak age of onset for AN and BN being 15.5 and 19.5 years for BED (Solmi et al. 2022). Adolescents may present to obesity treatment with undiagnosed EDs/disordered eating; hence it is imperative that tools are available to identify these adolescents early to support their access to tailored treatment pathways (Jebeile et al. 2021). Development and validation of ED questionnaires tailored to adolescents with higher weight remains a priority for future research.

Consistent with our original review, participants in the identified studies were largely female with limited reporting of race and ethnicity. When race and ethnicity were reported, studies often included predominantly White participants. This highlights a gap in our understanding of the diagnostic accuracy of the studied questionnaires in culturally diverse populations with higher weight. In addition, the diagnostic accuracy of several ED questionnaires which have either been validated for or developed in men has not been assessed in men with higher weight, for example, the Eating Pathology Symptoms Inventory (Forbush et al. 2013), Eating Disorder Assessment for Men (Stanford and Lemberg 2012), Eating for Muscularity Scale (Cooper et al. 2020) or Muscularity Oriented Eating Test (Murray et al. 2019). These tools incorporate aspects of eating pathology that may present more often in men, particularly muscularity oriented DEBs and body image concerns (Brown and Keel 2023), but their diagnostic accuracy in identifying EDs in men with higher weight remains unknown. It is important that future research prioritizes recruitment of culturally and gender diverse participants living with higher weight to ensure the range of screening questionnaires available are appropriate for patients from a variety of backgrounds (Halbeisen et al. 2022; Mittertreiner et al. 2024).

### 4.4 Strengths and Limitations

In this review, we systematically examined the literature published in the last 5 years to provide a timely update regarding the state of the evidence for ED questionnaire diagnostic accuracy in people with higher weight. The inclusion of studies examining ED questionnaire performance against a clinical diagnostic interview ensured the evidence reflects the diagnostic accuracy of questionnaires in relation to the gold standard for assessment of EDs. However, the completion of a clinical interview in community settings with large samples may be challenging, which may have limited the scope of the review, with most studies conducted in clinical settings. Seven of the included studies (examining the EDE‐Q, BES, QEWP‐R, NEQ, and EDO) were conducted in people seeking bariatric surgery, where there may be a risk of misreporting to avoid possible exclusion from treatment (Johnston et al. 2023). This may impact observed diagnostic accuracy of questionnaires used for screening and assessment in these settings. In addition, studies varied in the definitions used for ED diagnoses (dependent on the version of the DSM used) and DEBs. This heterogeneity may explain the variability in reported diagnostic accuracy between studies examining the same questionnaire. Finally, as discussed earlier, there was limited gender and cultural diversity in the included studies, thus the findings may not be generalizable to diverse populations living with higher weight.

## 5 Conclusion

In conclusion, this review examines developments in the diagnostic accuracy of ED questionnaires in adolescents and adults living with higher weight. Evidence regarding questionnaires that may be used to identify LOC eating in adolescents and adults, and binge eating and BED in adults with higher weight continues to emerge. However, there remains a paucity of evidence to inform clinical practice to identify the full range of EDs in adolescents and adults living with higher weight. Future research should prioritize the development of tailored questionnaires or examination of the diagnostic accuracy of existing questionnaires designed to assess EDs in adolescents and adults living with higher weight, with a focus on ensuring such tools are appropriate for diverse populations.

## Author Contributions

**Eve T. House:** conceptualization, methodology, investigation, formal analysis, project administration, writing – review and editing, writing – original draft, visualization. **Caitlin M. McMaster:** conceptualization, methodology, writing – review and editing, investigation. **Natalie B. Lister:** conceptualization, methodology, visualization, writing – review and editing. **Isabelle R. Jardine:** methodology, investigation, writing – review and editing. **Sasha J. Lorien:** methodology, investigation, writing – review and editing. **Anna Lene Seidler:** methodology, writing – review and editing, conceptualization. **Hiba Jebeile:** conceptualization, methodology, writing – review and editing, supervision.

## Funding

The authors have nothing to report.

## Disclosure

No generative AI tools were used in the study design, study execution, or manuscript preparation.

## Conflicts of Interest

The authors declare no conflicts of interest.

## Supporting information


**File S1:** Search strategy

**Table S1:** Summary of changes to the review protocol between the original review (search conducted September 2020) and the updated review (search conducted November 2025).
**Table S2:** Characteristics of studies included in the original and updated systematic review reporting on the diagnostic accuracy of eating disorder self‐report questionnaires against a clinical diagnostic interview in (a) adults and (b) adolescents. The table format and characteristics of studies and questionnaires included in the original review have been reproduced from the original publication (House et al. 2022).
**Table S3:** Risk of bias and applicability concerns in the included studies, assessed using the Quality Assessment of Diagnostic Accuracy Studies 2 (QUADAS‐2) (Whiting et al. 2011).

## Data Availability

The data that support the findings of this study are available from the corresponding author upon reasonable request.
