# Supplementary material for: Identifying Eating Disorders in Adolescents and Adults Living With Higher Weight: An Updated Systematic Review of Questionnaire Diagnostic Accuracy
Source: Int J Eat Disord. 2026 May 20;59(9):1876–96. doi: 10.1002/eat.70132 (PMC13536217; doi:10.1002/eat.70132)
Supplement: Supplementary file 1 [file EAT-59-1876-s001.docx]

**Table S1:** Summary of changes to the review protocol between the original review (search conducted September 2020) and the updated review (search conducted November 2025).

| **Review component** | **Original review** | **Updated review** | **Notes** |
| --- | --- | --- | --- |
| Search dates | Search conducted in September 2020, studies published from inception to the time of the search were eligible. | Search conducted in November 2025, the search was limited to studies published from 2020 onwards (in CINAHL the search was limited to September 2020 onwards). | As four of five databases did not allow searches to be limited to a specific month, studies published from January to September 2020 may have been captured in both searchers and represented twice in the PRISMA diagram (Figure 1). |
| Language | Studies published in a language other than English were excluded. | Studies published in a language other than English, identified in both the original and updated search, were translated using Google Translate to assess eligibility. | Of the 45 non-English studies excluded from the original review, 42 were able to be re-screened. Three were unable to be translated. None of the studies were eligible for inclusion.  In the updated search, two non-English studies were identified during full-text screening and translated, they did not meet the criteria for the review. |
| Contact with authors | Studies that included participants across the weight spectrum were only included if required data were reported separately for participants with higher weight. | Authors of studies identified in the updated search (i.e. published since 2020) were contacted if the studies reported measuring anthropometry and assessing diagnostic accuracy outcomes for participants across the weight spectrum. Studies were included if the authors provided required data for the subgroup of participants with higher weight. | Authors of thirteen papers were contacted, one of which provided subgroup data which was included in the review (Altman et al., 2020). |
| Data extraction | Data extraction was conducted in Microsoft Excel | Data extraction was conducted using the customisable data extraction form in Covidence systematic review software (Veritas Health Innovation, Melbourne, Australia, available at [www.covidence.org](http://www.covidence.org)). |  |
| Risk of bias | Consistent with recommendations in the first version of the Cochrane Handbook for Systematic Reviews of Diagnostic Test Accuracy (Deeks et al., 2013), risk of bias of included studies was assessed using the Quality Assessment of Diagnostic Accuracy Studies (QUADAS) tool (Whiting et al., 2003). | Consistent with recommendations in the second version of the Cochrane Handbook for Systematic Reviews of Diagnostic Test Accuracy (Deeks et al., 2023), risk of bias and applicability were assessed using the QUADAS-2 (Whiting et al., 2011). All included studies identified from the original and updated search were assessed using this tool. |  |

**Table S2:** Characteristics of studies included in the original and updated systematic review reporting on the diagnostic accuracy of eating disorder self-report questionnaires against a clinical diagnostic interview in **a)** adults and **b)** adolescents. The table format and characteristics of studies and questionnaires included in the original review have been reproduced from the original publication (House et al., 2022).

**a)**

| **Author (year), country, setting, sample size (n)^a^** | **Age (mean (SD) years); BMI (mean (SD) kg/m^2^); % female; race/ethnicity^b^ (%); SES** | **Index test version; cut-point; reference standard; diagnosis** | **Diagnostic accuracy** | | |
| --- | --- | --- | --- | --- | --- |
|  |  |  | **PPV/NPV** | **Sensitivity/Specificity** | **Other diagnostic accuracy outcomes** |
| ***Eating Disorder Examination Questionnaire (EDE-Q)***  Questionnaire adapted from the EDE (Cooper & Fairburn, 1987), assessing behavioural components of disordered eating across four domains: dietary restraint, eating concern, shape concern, and weight concern. Higher scores indicate greater psychopathology (Fairburn & Beglin, 1994). | | | | | |
| *Studies identified in review update* | | | | | |
| Everett et al. (2021), USA, clinical trial (setting unclear), n=94 | 51.8 (9.3) years; 37.3 (5.7) kg/m^2^; 80% female; 70% White; NR | 28-item EDE-Q; presence or absence of OBE; overeating section of EDE; OBE | 0.09/0.98 | 0.80/0.54 | NR |
| *Studies identified in 2022 review* | | | | | |
| Aardoom et al. (2012), The Netherlands, clinical (outpatient obesity program and day stay patients), n=433 (n=321 Group 1, n=112 Group 2) | **Group 1 (Obesity, no BED)** 41.1 (12.4) years; 41.9 (8.0) kg/m^2^; **Education Level (%)** 39% low, 40% intermediate, 21% high  **Group 2 (BED)** 36.7 (10.3) years; 38.7 (7.8) kg/m^2^; **Education Level (%)** 30% low, 43% intermediate, 26% high  **Whole sample^c^** 100% female; 93% Caucasian, 7% NR | 36-item EDE-Q; NR; standardized semi-structured interview based on DSM-IV; BED | NR | NR | **AUC** 0.72 (95% CI 0.67-0.77) |
| Hartmann et al. (2014)^d^, USA, clinical (hospital weight management centre), n=100 | 45.8 (12.0) years; 41.9 (9.1) kg/m^2^; 72% female; 81% White, 89% non-Hispanic/non-Latino; NR | EDE-Q version 6.0; score ≥4 on Q1, 2 or 3, Q10, Q11, 22 or 23, Q16, 17 or 18, and score = 0 on Q13 and 14; diagnostic interview for DSM-5 feeding and eating disorders; atypical AN | NR | NR | **Prevalence of atypical AN** interview 0%, EDE-Q 15% |
| Kalarchian et al. (2000), USA, clinical (gastric bypass, pre-operative), n=98 | 38.4 (10.7) years; 52.2 (10.1) kg/m^2^; 78% female; 89% White, 9% Black, 2% Hispanic; NR | 38-item EDE-Q; 2 binges per week; EDE (12^th^ edition); BE | 0.57/0.88 | 0.59/0.86 | NR |
| Mond et al. (2008)^d^, USA, community, n=64 | **Whole sample (n=257)**^e^ 27.6 (6.5) years; 28.1 (7.2) kg/m^2^; 100% female; 88% Caucasian, 5% Native American, 3% African American, 2% Hispanic, 1% Asian; **Education level** 95% completed high school, 32% associate’s or bachelor’s degree, 8% graduate school; **Employment** 39% full-time, 13% part-time, 28% full-time students, 11% caring for children, 4% unemployed and seeking employment. | 22-item EDE-Q (excluded behavioural questions); 3.1; EDE; ED (AN, BN, BED, EDNOS—DSM-IV criteria) | 0.42/NR | 0.77/0.73 | **AUC** 0.84 |
| Parker et al. (2016)^d^, Australia, clinical (bariatric surgery), n=117 | 43.8 (11.6) years; 42.5 (7.4) kg/m^2^; 79% female; NR; NR | 28-item EDE-Q; NR; EDE (16.0); BE (SBE, OBE, subthreshold BE) | NR | **OBE** 0.88/0.62 **SBE** 0.16/0.89 **Subthreshold BE** 0.20/0.85 | NR |
| Vander Wal et al. (2011)^d^, USA, community & university, n=41 | 52.0 (12.1) years; 39.1 (9.3) kg/m^2^; 71% female; 76% Caucasian/White, 20% African American/Black, 7% Hispanic/Latino/Latina; **Education level** graduated high school/ GED n=4, partial college n=8, graduated 2-year college n=7, graduated 4-year college n=8, partial graduate/ professional school n=3, completed graduate/ professional school n=11 | 39-item EDE-Q; restraint subscale ≥2.3–2.4 eating concern subscale ≥3.2 weight concern subscale ≥3.5 shape concern subscale ≥3.8 global score ≥3.2–3.3; EDE (12.0D); BED | **Restraint subscale** 0.37/0.64 **Eating concern subscale** 1.0/0.93 **Weight concern subscale** 0.68/0.91 **Shape concern subscale** 0.59/0.89 **Global score** 0.69/0.84 | **Restraint subscale** 0.40/0.62 **Eating concern subscale** 0.87/1.0 **Weight concern subscale** 0.87/0.77 **Shape concern subscale** 0.87/0.63 **Global score** 0.73/0.81 | NR |
| ***Binge-Eating Scale (BES)***  16-item self-report tool used to measure binge-eating behaviours, with higher scores indicating more severe binge-eating symptoms. A score of more than 17 indicates mild to moderate binge eating and score of 27 or above indicates severe binge eating (Gormally et al., 1982). | | | | | |
| *Studies identified in review update* | | | | | |
| Gormez et al. (2023), Türkiye, clinical (outpatient obesity clinic), n=188 | 45.9 (11.9) years; 36.3 (4.8) kg/m^2^; 76.5% female; NR; **Employment status** Employed 27%, Unemployed 9%, Housewife 46%, Student 10%, Retired 9%; **Education status** Illiterate 1%, Primary school 44%, Secondary-high school 39%, University 16% | BES (Turkish version); 18, SCID-5-CV; BED | 0.51/0.91 | 0.72/0.79 | **AUC** 0.79 (SD 0.04), (p<0.001) |
| Jeong et al. (2023), USA, clinical (academic medical hospital), n=1,133 (n=561 validation sample, n=572 replication sample) | **Validation sample** 46.2 (11.3) years; 48.8 (10.6) kg/m^2^; 70% female; 65% White, 23% black, 12% other; NR  **Replication sample** 45.8 (12.0) years; 49.4 (10.6) kg/m^2^; 75% female; 66% White, 23% black, 11% other; NR | BES; ≥15 and ≥17^f^; semi-structured clinical interview using DSM-5 criteria for BED; BED | **Validation sample**  **Cut-point ≥15** 0.48/0.91 **Cut-point ≥17** 0.52/0.89  **Replication sample**  **Cut-point ≥15** 0.47/0.87 **Cut-point ≥17** 0.49/0.83 | **Validation sample**  **Cut-point ≥15** 0.82/0.67 **Cut-point ≥17** 0.75/0.75  **Replication sample**  **Cut-point ≥15** 0.72/0.69 **Cut-point ≥17** 0.58/0.77 | **Validation sample**  **AUC** 0.80 (95% CI, 0.76-0.84) **Cut-point ≥15 *SR***^g^ 0.46 ***HR***^h^ 0.71 **Cut-point ≥17 *SR*** 0.39 ***HR*** 0.75  **Replication sample**  **AUC** 0.76 (95% CI, 0.72-0.80) **Cut-point ≥15 *SR*** 0.42 ***HR*** 0.70 **Cut-point ≥17 *SR*** 0.33 ***HR*** 0.72 |
| *Studies identified in 2022 review* | | | | | |
| Freitas et al. (2006), Brazil, clinical (participants in clinical trial of obesity treatment), n=178 | 36.4 (10.0) years; 36.3 (3.8) kg/m^2^; 100% female; NR; **Educational years** 74.7% participants with >12 years schooling | BES (Portuguese version); 17; SCID-I/P (DSM-IV); BED | 0.67/0.95 | 0.98/0.48 | NR |
| Grupski et al. (2013), USA, clinical (gastric bypass, pre-operative), n=473 | 41.7 (10.4) years; 50.5 (9.2) kg/m^2^; 85% female; NR; **Educational years** 13.7 (2.5) (range 7–25 years) | BES (not further specified); 17 and 27; SCID (for BED); BED | **Cut-point >17** 0.37/0.99 **Cut-point ≥27** 0.56/0.91 | **Cut-point >17** 0.94/0.76 **Cut-point ≥27** 0.37/0.96 | NR |
| Quilliot et al. (2019), France, clinical (bariatric surgery, pre-operative), n=340 | 41.7 (11.2) years; 46.3 (7.4) kg/m^2^; 78% female; NR; NR | BES (French version); 17; SCID-I/P (DSM-IV); BED | 0.55/0.71 | 0.51/0.75 | NR |
| Ricca et al. (2000)^d^, Italy, clinical (outpatient obesity treatment), n=344 | 43.5 (13.6) years; 35.8 (6.1) kg/m^2^; 83% female; NR; NR | 16-item BES; 17 and 27; SCID (DSM-III-R, with DSM-IV BED criteria); BED | **Cut-point >17** 0.26/0.98 **Cut-point ≥27** 0.57/0.96 | **Cut-point >17** 0.85/0.75 **Cut-point ≥27** 0.61/0.95 | NR |
| ***Questionnaire on Eating and Weight Patterns (QEWP/QEWP-R)***  28-item self-report questionnaire assessing binge eating and weight control behaviours (Spitzer et al., 1992). Positive screening result for BED if report≥2 binge-eating episodes without compensatory behaviours per week and significant distress associated with binge eating (Spitzer et al., 1992). | | | | | |
| *Studies identified in 2022 review* | | | | | |
| Borges et al. (2005), Brazil, clinical (treatment seeking for weight loss/binge eating), n=89 | **Participants with BED** 35.2 (10.8) years; 39.2 (11.8) kg/m^2^ **Participants without BED** 36.7 (11.8) years; 37.9 (6.6) kg/m^2^  **Whole sample** 100% female; 80% White; NR | QEWP-R (Portuguese version); NR; SCID-I/P; BED and BE | **BED** 0.79/0.56 **BE** 0.82/0.73 | **BED** 0.55/0.80 **BE** 0.88/0.63 | NR |
| Calugi et al. (2020), Italy, clinical (rehabilitative residential treatment program), n=604 | 53.1 (12.4) years; 41.6 (7.5) kg/m^2^; 65% female; NR; NR | QEWP-5 (Italian version); NR; EDE (Italian version); BED and BE (SBE, OBE) | **BED** 0.34/0.96 **OBE** 0.48/0.90 **SBE** 0.59/0.87 | **BED** 0.49/0.93 **OBE** 0.69/0.79 **SBE** 0.63/0.86 | NR |
| De Zwaan et al. (1993), USA/Austria, clinical (not further specified), n=100 | 39.2 (NR) years; 35.9 (NR) kg/m^2^; 100% female; NR; NR | QEWP (1992); NR; SCID (DSM-III-R version with proposed DSM-IV criteria for BED); BED | 0.78/0.80 | 0.72/0.84 | NR |
| Dymek-Valentine et al. (2004) , USA, clinical (gastric bypass clinic, pre-operative), n=168 | 39.5 (9.3) years; 50.8 (9.2) kg/m^2^; 85% female; 59% Caucasian, 26% African American, 11% Hispanic, 4% other; **Education level** NR, no difference between education level of those with and without BED | 28-item QEWP-R; NR; ED-SCID (DSM-IV); BED and partial BED | **BED** 0.40/0.94 **BED or partial BED** 0.55/0.88 | **BED** 0.73/0.80 **BED or partial BED** 0.79/0.70 | NR |
| Hartmann et al. (2014)^d^, USA, clinical (hospital weight management center), n=100 | 45.8 (12.0) years; 41.9 (9.1) kg/m^2^; 72% female; 81% White, 89% non-Hispanic/non-Latino; NR | 28-item QEWP-R; BN = score =1 on Q10, 11 and 12 with frequency ≥3, score ≥3 on Q17, Score = 1 on Q18, 19, 20, 21, 22, or 23 with frequency ≥2 on Q18a, 19a, 20a, 21a, 22a, or 23a; BED = score = 1 on Q10, 11 and 12 with frequency ≥3, score ≥3 on Q13, score ≥2 on Q15 or 16; subthreshold BN = criteria for BN with lower frequency (Q12 frequency of 1 or 2); subthreshold BED = criteria for BED with lower frequency (Q12 frequency of 1 or 2); purging disorder = score = 1 on Q18, 19 or 20 and frequency ≥2 on Q18a, 19a, 20a score ≥3 on Q17 and score = 0 on Q10 and 11; SCID-IV and diagnostic interview for DSM-5 feeding and eating disorders; BN, BED and OSFED (subthreshold BED, subthreshold BN, purging disorder) | NR | **BN/BED (DSM-IV)** 0.21/0.97 **BN (DSM-IV)** 0.50/0.00 **BED (DSM-IV)** 0.71/0.96 **BN/BED (DSM-5)** 0.5-0.56/0.98-1.00 **Purging disorder (DSM-5)** 1.0/0.01 **Subthreshold BED (DSM-5)** NR/1.0 | **AUC *BN/BED (DSM-IV)*** 0.59 ***BN (DSM-IV)*** 0.75 ***BED (DSM-IV)*** 0.84  **Prevalence of ED *BN (DSM-IV)*** 2% interview; 1% QEWP-R ***BED (DSM-IV)*** 9% interview; 7% QEWP-R ***BN (DSM-5)*** 2% interview; 1% QEWP-R ***BED (DSM-5)*** 9% interview; 7% QEWP-R ***Subthreshold BED (DSM-5)*** 5% interview; 1% QEWP-R ***Subthreshold BN (DSM-5)*** 2% interview; 0% QEWP-R ***Purging disorder (DSM-5)*** 1% interview; 1% QEWP-R |
| Parker et al. (2016)^d^, Australia, clinical (bariatric surgery), n=101 | 43.8 (11.6) years; 42.5 (7.4) kg/m^2^; 79% female; NR; NR | 28-item QEWP-R; NR; EDE (16.0); BE (SBE, OBE, subthreshold BE) | NR | **OBE** 0.71/0.64 **SBE** 0.25/0.86 **Subthreshold BE** 0.07/0.88 | NR |
| ***SCOFF questionnaire***  Brief screening questionnaire consisting of five “yes/no” questions which can usually be completed in 30 seconds. A positive response (yes) to ≥2 questions is generally used to identify risk of ED (Mond et al., 2008; Morgan et al., 1999). | | | | | |
| *Studies identified in 2022 review* | | | | | |
| Liu et al. (2015), Taiwan, clinical (psychiatric clinic), n=178 | 30.5 (7.8) years; BMI ≥27kg/m^2^; 49% female; NR; **Educational years** men 14.5 (2.5), women 14.3 (2.6) | M-SCOFF (Mandarin Chinese version); 2 (males) 3 (females); SCID-I/P (DSM-IV); ED (AN, BN, BED, EDNOS) | NR | **Males** 0.67/0.65 **Females** 0.96/0.83 | **AUC** ***Males*** 0.66 ***Females*** 0.90 |
| Mond et al. (2008)^d^, USA, community, n=64 | **Whole sample (n=257)**^e^  27.6 (6.5) years; 28.1 (7.2) kg/m^2^; 100% female; 88% Caucasian, 5% Native American, 3% African American, 2% Hispanic, 1% Asian; **Education level** 95% completed high school, 32% associate or bachelor’s degree, 8% graduate school; **Employment** 39% full-time, 13% part-time, 28% full-time students, 11% caring for children, 4% unemployed and seeking employment. | SCOFF; 2; EDE; ED (AN, BN, BED, EDNOS—DSM-IV criteria) | 0.3/NR | 0.69/0.59 | **AUC** 0.72 |
| Solmi et al. (2015), UK, community, n=77 | **Whole interview sample (n=145)**^I^ NR (range 16-90 years); NR n=46 with overweight, n=31 with obesity; 75% female; 57% White, 29% Black, 3% Asian, 11% other; **Education level** 10% no qualification, 48% GCSE/A-level, 42% degree level or above | SCOFF; 2; SCID-I/NP (DSM-IV); ED | **Overweight** 0.43/1.0 **Obesity** 0.6/0.88 **Overweight and obesity** 0.50/0.95 | **Overweight** 1.0/0.64 **Obesity** 0.82/0.70 **Overweight and obesity** 0.90/0.66 | NR |
| ***Night Eating Questionnaire (NEQ) and Night Eating Question***  The NEQ is 14-item tool to assess the severity of night eating syndrome and its psychological and behavioural symptoms. Possible scores range from 0 to 52 with higher scores indicating greater NES symptoms (Allison et al., 2008). Vander Wal et al. (2005) used a single Night Eating Question to determine entry into the study followed by administration of the first nine items of the NEQ, as they appear in the Weight and Lifestyle Inventory (Wadden & Foster, 2006). The single question “To what extent does snacking after dinner contribute to your weight problem,” was scored on a 5-point scale, participants with a score ≥4 were recruited (Vander Wal et al., 2005). In the study (Vander Wal et al., 2005), the items from the WALI were referred to as the Night Eating Syndrome Questionnaire (NESQ), as these are taken from the NEQ, in this review they are considered together with other studies using the NEQ. | | | | | |
| *Studies identified in 2022 review* | | | | | |
| Allison et al. (2008), USA, clinical (bariatric surgery candidates, preoperative), n=194 | 44.0 (10.7) years; 50.4 (8.0) kg/m^2^; 83% female; 69% Caucasian, 25% African American, 6% other; NR | 14-item NEQ; 25 and 30; NESHI; NES | **Cut-point ≥25** 0.41/0.95 **Cut-point ≥30** 0.73/0.94 | NR | NR |
| Hartmann et al. (2014)^d^, USA, clinical (hospital weight management centre), n=100 | 45.8 (12.0) years; 41.9 (9.1) kg/m^2^; 72% female; 81% White, 89% non-Hispanic/non-Latino; NR | 14-item NEQ; 30; diagnostic interview for DSM-5 feeding and eating disorders; NES | NR | <0.01/NR | **Prevalence of NES** 6% interview, 4% NEQ |
| Vander Wal et al. (2005)^j^, USA, Clinical (RCT participants), n=59 | 50.7 (10.4) years; 35.3 (7.4) kg/m^2^; 76% female; NR; NR | Night Eating Question and 9-items of the NEQ (from the WALI); 4 for Night Eating Question, 5–10 for NEQ to optimise sensitivity^k^; structured clinical interview for NES; NES | **Night Eating Question** 0.16-0.98/0.25-0.87 **NEQ** 0.23-0.95/1.0 | **Night Eating Question** 0.80-0.98/0.11-0.67 **NEQ** 1.0/0.00-0.40 | NR |
| ***Bulimic Investigatory Test, Edinburgh (BITE)***  33-item self-rating scale to assess symptoms and severity of binge eating and purging behaviour (Henderson & Freeman, 1987). The possible score ranged from 0 to 30 (Ricca et al., 2000). A score of 10–19 indicates abnormal eating behaviour and score of 20 or above indicates highly disordered eating patterns and presence of binge eating. | | | | | |
| *Studies identified in 2022 review* | | | | | |
| Orlandi et al. (2005), Italy, clinical (outpatient obesity treatment), n=388 | 40.3 (13.2) years; 35.2 (6.0) kg/m^2^; 81% female; NR; NR | BITE (Italian version); 10 and 20 on the symptom sub-scale; EDE-12.0D; BED | **Cut-point ≥10** 0.14/0.99 **Cut-point ≥20** 0.30/0.95 | **Cut-point ≥10** 0.93/0.55 **Cut-point ≥20** 0.41/0.92 | NR |
| Ricca et al. (2000)^d^, Italy, clinical (outpatient obesity treatment), n=344 | 43.5 (13.6) years; 35.8 (6.1) kg/m^2^; 83% female; NR; NR | BITE (1987); 10 and 20; SCID (DSM-IV criteria for BED); BED | **Cut-point ≥10** 0.72/0.98  **Cut-point ≥20** 0.31/0.93 | **Cut-point ≥10** 0.91/0.51 **Cut-point ≥20** 0.33/0.92 | NR |
| ***Clinical Impairment Assessment (CIA)***  The CIA is a 16-item questionnaire assessing psychosocial impairment associated with eating disorders across three domains—personal, cognitive, and social (Bohn et al., 2008). | | | | | |
| *Studies identified in 2022 review* | | | | | |
| Hartmann et al. (2014)^d^, USA, clinical (hospital weight management center), n=100 | 45.8 (12.0) years; 41.9 (9.1) kg/m^2^; 72% female; 81% White, 89% non-Hispanic/non-Latino; NR | 16-item CIA; 16; SCID-IV and diagnostic interview for DSM-5 feeding and eating disorders; any ED (BN, BED, and OSFED) according to DSM-IV and DSM-5 criteria | **ED (DSM-IV)** 0.20/0.94 **ED (DSM-5)** 0.51/0.79 | **ED (DSM-IV)** 0.64/0.69 **ED (DSM-5)** 0.59/0.74 | NR |
| ***Binge-Eating Disorder Screener-7 (BEDS-7)***  7-item self-report screening tool to identify risk of BED. To receive a positive BED result, participants must answer “yes” to the first two questions relating to overeating and distress related to this. They must answer “sometimes,” “often,” or “always” to Q3-6 and “sometimes” or “rarely/never” to Q7 (related to purging) (Herman et al., 2016). | | | | | |
| *Studies identified in 2022 review* | | | | | |
| Herman et al. (2016), USA, community, n=80 | **True positives (n=16, 1 with normal weight)** 42.4 (12.0) years; 36.3 (9.4) kg/m^2^  **False positives (n=46, 9 with normal weight)** 36.4 (10.6) years; 32.4 (8.4) kg/m^2^  **True negatives (n=29, 4 with normal weight)** 47.0 (11.4) years; 31.4 (7.9) kg/m^2^  **Whole sample** 60% female; 68% White, 14% Black, 7% Asian, 6% Hispanic or Latino, 4% other; NR | BEDS-7; cut-point not specified; modified SCID-I/NP; BED | **BMI 25–29.9** 0.20/1.00 **BMI 30–39.9** 0.27/1.00 **BMI ≥40** 0.45/1.00 **BMI ≥25** 0.29/1.00 | **BMI 25–29.9** 1.00/0.48 **BMI 30–39.9** 1.00/0.34 **BMI≥40** 1.00/0.40 **BMI≥25** 1.00/0.40 | NR |
| ***Eating Disorders in Obesity questionnaire (EDO)***  11-item questionnaire designed to screen for DSM-IV ED (de Man Lapidoth et al., 2007). Adapted from the Survey for Eating Disorders (SED) (Ghaderi & Scott, 2002) for use in weight management therapy settings. Eight of 11 items only relevant to patients reporting binge eating. “Binge eaters” (BE) reported out-of-control objective binge-eating episodes. Participants that met criteria for BN, BED, or EDNOS were classified as ED. | | | | | |
| *Studies identified in 2022 review* | | | | | |
| de Man Lapidoth et al. (2007), Sweden, clinical (surgical and behavioural obesity clinics), n=97 | 41.1 (10.6) years; 44.2 (7.7) kg/m^2^; 72% female; NR; NR | 11-item EDO; NR; EDE (AN questions excluded, BED described as per DSM-IV); ED and BE | **ED** 0.90/0.97 **BE** 0.72/0.90 | **ED** 0.75/0.99 **BE** 0.82/0.83 | NR |
| ***Risk Factors for Binge-Eating Disorder in Overweight (REO)***  30-item screening questionnaire designed to identify individuals with obesity who are at risk of BED. Each item is rated on a scale of 1–5 (1 = never; 5 = almost always) with a total score ranging from 30 to 150, with higher scores indicating more symptoms of BED (Wever et al., 2018). | | | | | |
| *Studies identified in 2022 review* | | | | | |
| Wever et al. (2018), The Netherlands, clinical (psychology and nutrition practices), n=50 | **No BED (n=27)** 53.2 (12.5) years; 35.5 (6.5) kg/m^2^; 78% female; NR; NR  **BED (n=23)** 37.0 (12.6) years; 36.8 (6.7) kg/m^2^; 83% female; NR; NR | 30-item REO; 83.5; EDE not further specified; BED | 0.07/0.03 | 0.95/0.82 | **AUC** 0.89 |
| ***Bulimia Test-Revised (BULIT-R)***  36-item questionnaire (with 28 scored questions), originally designed to identify bulimic symptoms according to the DSM-III. Scores range from 28 to 140, with higher scores indicating more bulimic symptomatology (Thelen et al., 1991). | | | | | |
| *Studies identified in 2022 review* | | | | | |
| Vander Wal et al. (2011)^d^, community and university, n=41 | 52.0 (12.1) years; 39.1 (9.3) kg/m^2^; 71% female; 20% African American/Black, 76% Caucasian/White, 7% Hispanic/Latino/Latina; **Education level** graduated high school/ GED n=4, partial college n=8, graduated 2-year college n=7, graduated 4-year college n=8, partial graduate/ professional school n=3, completed graduate/ professional school n=11 | 36-item BULIT-R; 80; EDE (12.0D); BED | 0.94/1.0 | 1.0/0.96 | NR |
| ***The Binge-Eating Disorder Test (BEDT)***  23-item questionnaire that is derived from the items related to binge-eating disorder symptoms of the BULIT-R (Thelen et al., 1991) | | | | | |
| *Studies identified in 2022 review* | | | | | |
| Vander Wal et al. (2011)^d^, community and university, n=41 | 52.0 (12.1) years; 39.1 (9.3) kg/m^2^; 71% female; 20% African American/Black, 76% Caucasian/White, 7% Hispanic/Latino/Latina; **Education level** graduated high school/ GED n=4, partial college n=8, graduated 2-year college n=7, graduated 4-year college n=8, partial graduate/ professional school n=3, completed graduate/ professional school n=11 | 23-item BEDT; 75; EDE (12.0D); BED | 1.0/1.0 | 1.0/1.0 | NR |
| ***LOC/binge eating screening tool***  The LOC/binge eating screening tool was developed in the included study (Manasse et al., 2021) and included 15 items assessing eating pathology in the last three months. The tool included three categories of items: 1) eating disorder behaviours, 2) binge-eating features and distress from binge-eating (based on DSM-5 criteria), and 3) general overeating behaviours. One item examining distress was rated on a Likert scale, all other items were ‘yes or no’ questions. Four of the behavioural items were adapted from the EDE (Cooper & Fairburn, 1987; Manasse et al., 2021). | | | | | |
| *Studies identified in review update* | | | | | |
| Manasse et al. (2021), USA, clinical research centre, n=364 | 50.0 (13.9) years; 35.7 (7.7) kg/m^2^; 76% female; NR; NR | LOC/binge eating screening tool; NR; EDE binge eating module; LOC eating and full threshold BE^l^ | NR | **LOC eating** 0.78/0.77 **Females** 0.79/0.76 **Males** 0.72/0.79 **Leave-one-out cross validation** 0.75/0.74 ***Females*** 0.77/0.75 ***Males*** 0.62/0.58 **Full-threshold BE** 0.75/0.74 **Females** 0.73/0.73 **Males** 0.85/0.82 **Leave-one-out cross validation** 0.74/0.70 ***Females*** 0.71/0.70 ***Males*** 0.64/0.71 | **LOC eating Canonical correlation between LOC status and self-report-items** 0.59 **Derived discriminant function** χ^2^ = 154.89, df = 16, *P* < 0.01  **Full-threshold BE Canonical correlation between full-threshold and non-full-threshold groups and self-report-items** 0.52 **Derived discriminant function** χ^2^ = 110.77, df = 16, *P* < 0.01 |
| ***Combination of the EDE-Q, QEWP-R and NEQ***  These questionnaires have been described above. | | | | | |
| *Studies identified in 2022 review* | | | | | |
| Hartmann et al. (2014)^d^, USA, clinical (hospital weight management center), n=100 | 45.8 (12.0) years; 41.9 (9.1) kg/m^2^; 72% female; 81% White, 89% non-Hispanic/non-Latino; NR | 28-item QEWP-R and EDEQ version 6.0 and 14-item NEQ; cut-points specified above; diagnostic interview for DSM-5 feeding and eating disorders; any ED (BN, BED and OSFED) | NR | 0.47/0.78 | **AUC** 0.62 |

**b)**

| **Author (year), country, setting, sample size (n)^a^** | **Age (mean [SD] years); BMI (mean [SD] kg/m^2^); % female; race/ethnicity^b^ (%); SES** | **Index test version; cut-point; reference standard; diagnosis** | **Diagnostic accuracy** | | |
| --- | --- | --- | --- | --- | --- |
|  |  |  | **PPV/NPV** | **Sensitivity/Specificity** | **Other diagnostic accuracy outcomes** |
| ***Children’s Eating Disorder Examination Questionnaire and Youth Eating Disorder Examination Questionnaire (ChEDE-Q and YEDE-Q)***  ChEDE-Q and YEDE-Q are adaptations of the EDE-Q for use in children and adolescents. Modifications include simplification of descriptive terms and addition of images and vignettes to help young people understand behaviors described in the questionnaire. Higher scores indicate greater psychopathology (Decaluwé & Braet, 2004; Goldschmidt et al., 2007) | | | | | |
| *Studies identified in 2022 review* | | | | | |
| Decaluwé and Braet (2004), Belgium, clinical (inpatient obesity treatment), n=139 | 12.8 (1.8) years; adjusted BMI 172.3 (25.6)%; 58% female; NR; NR | ChEDE-Q (Dutch version); NR; ChEDE; BE | 0.22/0.97 | 0.79/0.68 | NR |
| Goldschmidt et al. (2007), USA, online, n=35 | 13.8 (1.6) years; 35.3 (7.1) kg/m^2^; 71% female; 51% Caucasian, 40% African American, 3% Hispanic, 6% other; NR | YEDE-Q (adapted from EDE-Q 5.2); NR; ChEDE 12.0; BE | 1.0/0.9 | 0.57/1.0 | NR |
| Goossens and Braet (2010), Belgium, clinical (inpatient weight management) and community (nontreatment seeking), n=235 | 14.3 (1.5) years; adjusted BMI 160.6 (29.6)%; 56% female; NR; NR | ChEDE-Q (Dutch version); NR; ChEDE; disordered eating behaviors | NR | NR | **% concordant/ non-concordant cases *Objective overeating*** 52.56/47.44 ***OBE*** 68.51/31.49 ***SBE*** 66.38/33.62 ***Self-induced vomiting*** 85.96/14.04 ***Laxative/diuretic misuse*** 84.68/15.32 ***Appetite depressant misuse*** 84.26/15.74 ***Excessive exercise*** 68.94/31.06 |
| ***Adolescent Binge-Eating Disorder Questionnaire (ADO-BED)***  French language, 10-item, questionnaire to assess binge-eating behaviour in adolescents with obesity. Q1–3 are yes/no questions relating to binge-eating behaviour, Q4 and 5 relate to frequency of the behaviours and Q6 is a yes/no question relating to purging behaviour (Chamay-Weber et al., 2017) | | | | | |
| *Studies identified in 2022 review* | | | | | |
| Chamay-Weber et al. (2017), Switzerland, clinical (paediatric obesity care centre), n=94 | Median (range) 14 (11–18) years; BMI >97^th^ percentile for age and sex; 60% female; NR; NR | ADO-BED (French version); NR (diagnostic accuracy reported by question); SCID (BED portion of French version); BED | **Q1** 0.31/1.0  **Q2** 0.36/0.93  **Q1 or 2** 0.28/1.0  **Q3a** 0.43/0.75  **Q3b** 0.47/0.75 **Q3c** 0.35/0.78 **Q3d** 0.36/1.0 **Q3e** 0.44/0.87 **Q4** 0.65/0.80 **Q5** 0.33/0.73 **Q6** 0.29/0.69 | **Q1** 1.0/0.36 **Q2** 0.86/0.56  **Q1 or 2** 1.0/0.27 **Q3a** 0.43/0.75 **Q3b** 0.38/0.81 **Q3c** 0.76/0.38 **Q3d** 1.0/0.21 **Q3e** 0.81/0.54 **Q4** 0.52/0.87 **Q5** 0.81/0.24 **Q6** 0.33/0.65 | NR |
| ***Children’s Brief Binge-Eating Questionnaire (CBBEQ)***  7-item tool based on the Children's Binge-Eating Disorder Scale (C-BEDS) (Shapiro et al., 2007). The first six items consist of “yes/no” questions and the seventh question asks about the onset of behavior. A cut-off score ≥8 was used to identify individuals at risk of BED (Franklin et al., 2019). | | | | | |
| *Studies identified in 2022 review* | | | | | |
| Franklin et al. (2019), USA, clinical (obesity clinic), n=70 | 13.6 (2.7) years; 36.9 (8.5) kg/m^2^; 57% female; 64% Caucasian, 27% African American, 8% other; 63% Hispanic; **Insurance type** 67% Medicaid, 19% private insurance, 14% Children's Health Insurance Program | CBBEQ (adapted from C-BEDS); 8; EDA (for DSM-5 BED); BED | NR | 1.0/0.93 | **PLR** 0.33 **NLR** 1.0 |
| ***Loss of Control Eating Disorder Questionnaire (LOC-ED-Q)***  This questionnaire was designed by the authors of the included study (Altman et al., 2020). It consists of 19-items which assess the presence and frequency of LOC-eating. The questionnaire was intended to have the ability to make a provisional diagnosis of LOC-ED, it was designed to be comprehensible for children (Altman et al., 2020). | | | | | |
| *Studies identified in review update* | | | | | |
| Altman et al. (2020)^d^, USA, community (longitudinal non-interventional study), n=65^m^ | NR (range 10-17 years); NR (all BMIz >1); 60% female; 34% non-Hispanic White, 43% non-Hispanic Black, 23% other; NR | LOC-ED-Q; presence of ≥1 LOC-eating episode in past three months; EDE or ChEDE (for participants <13 years or those requiring additional clarification); LOC eating | 0.50/0.87 | 0.42/0.91 | **Diagnostic accuracy (efficiency)**^n^ 0.82 |
| ***Questionnaire on Eating and Weight Patterns-5 Children/Adolescent (QEWP-C-5)***  This is an adaption of the QEWP-5 for children and adolescents (Yanovski et al., 2015). It was developed from an earlier version, the QEWP-A (Johnson et al., 1999), and consists of 32-items which are designed to assess for the presence of BED, including items to examine LOC-eating over the past three months (Altman et al., 2020). | | | | | |
| *Studies identified in review update* | | | | | |
| Altman et al. (2020)^d^, USA, community (longitudinal non-interventional study), n=65^m^ | NR (range 10-17 years); NR (all BMIz >1); 60% female; 34% non-Hispanic White, 43% non-Hispanic Black, 23% other; NR | QEWP-C-5; presence of ≥1 LOC-eating episode in past three months; EDE or ChEDE (for participants <13 years or those requiring additional clarification); LOC eating | 0.56/0.88 | 0.42/0.93 | **Diagnostic accuracy (efficiency)** 0.83 |
| ***Combination of LOC-ED-Q and QEWP-C-5***  These questionnaires have been described above. | | | | | |
| *Studies identified in review update* | | | | | |
| Altman et al. (2020)^d^, USA, community (longitudinal non-interventional study), n=65^m^ | NR (range 10-17 years); NR (all BMIz >1); 60% female; 34% non-Hispanic White, 43% non-Hispanic Black, 23% other; NR | QEWP-C-5 and LOC-ED-Q; presence of ≥1 LOC-eating episode in past three months; EDE or ChEDE (for participants <13 years or those requiring additional clarification); LOC eating | 0.46/0.89 | 0.50/0.87 | **Diagnostic accuracy (efficiency)** 0.80 |

*Abbreviations:* ADO-BED – Adolescent Binge-Eating Disorder Questionnaire, AN – anorexia nervosa, AUC – area under the curve, BE – binge eating, BED – binge-eating disorder, BEDS-7 – Binge-Eating Disorder Screener-7, BEDT – Binge-Eating Disorder Test, BES – Binge Eating Scale, BITE – Bulimic Investigatory Test Edinburgh, BMI – body mass index, BULIT-R – Bulimia Test-Revised, CBBEQ – Children’s Brief Binge Eating Questionnaire, C-BEDS – Children’s Binge-Eating Disorder Scale, ChEDE – Children’s Eating Disorder Examination, ChEDE-Q – Children’s Eating Disorder Examination Questionnaire, CIA – Clinical Impairment Assessment, DSM-III-R – Diagnostic and Statistical Manual of Mental Disorders-Revised 3rd edition, DSM-IV – Diagnostic and Statistical Manual of Mental Disorders Fourth Edition, DSM-5 – Diagnostic and Statistical Manual of Mental Disorders Fifth Edition, EDA – Eating Disorder Assessment, EDE – Eating Disorder Examination, EDE-Q – Eating Disorder Examination Questionnaire, EDO – Eating Disorders in Obesity questionnaire, HR – hit rate, LOC – loss of control, NEQ – Night Eating Questionnaire, NES – Night Eating Syndrome, NESHI – Night Eating Syndrome History and Inventory, OBE – objective binge eating, NLR – negative likelihood ratio, NPV – negative predictive value, NR – not reported, OSFED – other specified feeding and eating disorder, PLR – positive likelihood ratio, PPV – positive predictive value, QEWP – Questionnaire on Eating and Weight Patterns, QEWP-R – Questionnaire on Eating and Weight Patterns-Revised, REO – Risk Factors for Binge-Eating in Overweight, SBE – subjective binge eating, SCID – Structured Clinical Interview based on the DSM, SCID-5-CV – Structured Clinical Interview for DSM-5-Disorders - Clinician Version, SCID-I/P– Structured Clinical Interview based on the DSM - Patient version, SD – standard deviation, SE – standard error, SR – selection ratio, USA – United States of America, WALI – Weight and Lifestyle Inventory, YEDE-Q – Youth Eating Disorder Examination Questionnaire

a. The sample size reported reflects the number of participants from the study for which data relevant to this review is available.
b. The terms used to describe race or ethnicity are the same as those in the original publications
c. In this study, ethnicity was only reported for the whole study sample, this included n=1,491 females (of which only samples with BED and obesity were included in this review)
d. These studies appear more than once in the table as they validated more than one questionnaire
e. In this study, diagnostic accuracy outcomes are presented for 64 participants with obesity who completed the questionnaires and interview, demographic data was only reported for the whole sample of 257 participants (across the weight spectrum), of whom 147 completed an interview. In this study, the SCOFF was modified for use in the United States
f. This study examined the diagnostic accuracy of the BES across a range of cut-points from 10 to 20, the results for cut-off scores of 15 and 17 are reported in the table as authors identified these as the optimal cut-off score to balance sensitivity and specificity and the traditional cut-off score, respectively.
g. Selection ratio refers to the proportion of the sample that is predicted to have BED at the associated BES cut-off
h. Hit rate refers to the proportion of correct predictions in the overall sample
i. In this study, diagnostic accuracy outcomes are presented for 77 participants with overweight and obesity who completed the questionnaire and interview, demographic data was only reported for the sample of 145 participants (across the weight spectrum) who completed the questionnaire and interview
j. Diagnostic accuracy and cut-off values presented as a range as this varied dependent on which of six definitions of the Night Eating Syndrome were used: (a) eating more in the evening than any other time of day; (b) eating at least 50% of one's daily caloric intake after 7 p.m.; (c) eating more in the evening than any other time of day and no morning appetite; (d) eating at least 50% of one's daily caloric intake after 7 p.m. and no morning appetite; (e) eating more in the evening than any other time of day, no morning appetite, and a sleep disturbance; (f) eating at least 50% of one's daily caloric intake after 7 p.m., no morning appetite, and sleep disturbance.
k. Cut-points of 11-21 were also examined in this study to optimize specificity, data not shown in table
l. In this study, participants were categorised as having “LOC eating” if the endorsed any loss-of-control eating over the past three months in the clinical interview, they were classified as having “full threshold binge eating” if they reported at least 12 objective binge eating episodes in the past three months in the clinician interview.
m. The study reported on findings for 218 children and adolescents, data included in the table was provided by study authors for the subset of adolescents with BMI z-score >1
n. Defined as the proportion of true positives and true negatives out of all participants

**Table S3:** Risk of bias and applicability concerns in the included studies, assessed using the Quality Assessment of Diagnostic Accuracy Studies 2 (QUADAS-2) (Whiting et al., 2011).

|  | **Risk of bias** | | | | **Applicability Concerns** | | |
| --- | --- | --- | --- | --- | --- | --- | --- |
| **Study (Author(year))** | **Domain 1: Patient selection** | **Domain 2: Index test(s)** | **Domain 3: Reference standard** | **Domain 4: Flow and timing** | **Domain 1: Patient selection** | **Domain 2: Index test(s)** | **Domain 3: Reference standard** |
| **Adults** | | | | | | | |
|  | **EDE-Q** | | | | | | |
| Aardoom et al. (2012) | High | Unclear | Unclear | Unclear | Low | Low | Low |
| Everett et al. (2021) | High | Unclear | Unclear | Low | Low | Low | Low |
| Hartmann et al. (2014)^a^ | Low | Unclear | Low | Unclear | Low | Low | Low |
| Kalarchian et al. (2000) | Low | Unclear | Unclear | High | Low | Low | Low |
| Mond et al. (2008)^a^ | Low | High | Unclear | High | Low | Low | Low |
| Parker et al. (2016)^a^ | Low | Unclear | Unclear | High | Low | Low | Low |
| Vander Wal et al. (2011)^a^ | High | High | Low | Unclear | Low | Low | Low |
|  | **QEWP/QEWP-R** | | | | | | |
| Borges et al. (2005) | Low | Low | Low | High | Low | Low | Low |
| Calugi et al. (2020) | Low | Unclear | Unclear | High | Low | Low | Low |
| De Zwaan et al. (1993) | Unclear | Low | Low | High | Low | Low | Low |
| Dymek-Valentine et al. (2004) | Low | Low | Unclear | High | Low | Low | Low |
| Hartmann et al. (2014)^a^ | Low | Unclear | Low | Unclear | Low | Low | Low |
| Parker et al. (2016)^a^ | Low | Unclear | Unclear | High | Low | Low | Low |
|  | **BES** | | | | | | |
| Freitas et al. (2006) | High | Low | Low | Unclear | Low | Low | Low |
| Gormez et al. (2023) | Low | High | Low | High | Low | Low | Low |
| Grupski et al. (2013) | Low | Unclear | Unclear | Low | Low | Low | Low |
| Jeong et al. (2023) | Unclear | Unclear | High | Unclear | Low | Low | Low |
| Quilliot et al. (2019) | Low | Low | Unclear | Unclear | Low | Low | Low |
| Ricca et al. (2000)^a^ | Low | Unclear | Unclear | Unclear | Low | Low | Low |
|  | **SCOFF** | | | | | | |
| Liu et al. (2015) | Low | High | Low | Low | Low | Low | Low |
| Mond et al. (2008)^a^ | Low | High | Unclear | High | Low | Low | Low |
| Solmi et al. (2015) | High | Low | Unclear | High | Low | Low | Low |
|  | **NEQ and Night Eating Question** | | | | | | |
| Allison et al. (2008) | Low | Unclear | High | High | Low | Low | Low |
| Hartmann et al. (2014)^a^ | Low | Unclear | Low | Unclear | Low | Low | Low |
| Vander Wal et al. (2005) | High | High | High | High | Low | Low | Low |
|  | **BITE** | | | | | | |
| Orlandi et al. (2005) | Low | Unclear | Unclear | Unclear | Low | Low | Low |
| Ricca et al. (2000)^a^ | Low | Unclear | Unclear | Unclear | Low | Low | Low |
|  | **CIA** | | | | | | |
| Hartmann et al. (2014)^a^ | Low | Unclear | Low | Unclear | Low | Low | Low |
|  | **BEDS-7** | | | | | | |
| Herman et al. (2016) | High | High | Low | High | Low | Low | Low |
|  | **EDO** | | | | | | |
| de Man Lapidoth et al. (2007) | High | Low | High | High | Low | Low | Low |
|  | **REO** | | | | | | |
| Wever et al. (2018) | High | High | Low | High | Low | Low | Low |
|  | **BULIT-R** | | | | | | |
| Vander Wal et al. (2011)^a^ | High | High | Low | Unclear | Low | Low | Low |
|  | **BEDT** | | | | | | |
| Vander Wal et al. (2011)^a^ | High | High | Low | Unclear | Low | Low | Low |
|  | **LOC/binge eating screening tool** | | | | | | |
| Manasse et al. (2021) | Low | High | Unclear | High | Low | Low | Low |
|  | **QEWP-R, EDE-Q and NEQ** | | | | | | |
| Hartmann et al. (2014)^a^ | Low | Unclear | Low | Unclear | Low | Low | Low |
| **Adolescents** | | | | | | | |
|  | **ChEDE-Q/YEDE-Q** | | | | | | |
| Decaluwé and Braet (2004) | Low | Low | Low | Unclear | Low | Low | Low |
| Goldschmidt et al. (2007) | High | Low | Low | High | Low | Low | Low |
| Goossens and Braet (2010) | Unclear | Unclear | Unclear | Unclear | Low | Low | Low |
|  | **ADO-BED** | | | | | | |
| Chamay-Weber et al. (2017) | Low | Unclear | Low | High | Low | Low | Low |
|  | **CBBEQ** | | | | | | |
| Franklin et al. (2019) | High | High | Unclear | High | Low | Low | Low |
|  | **LOC-ED-Q** | | | | | | |
| Altman et al. (2020)^a^ | Low | Low | Unclear | Unclear | Low | Low | Low |
|  | **QEWP-C-5** | | | | | | |
| Altman et al. (2020)^a^ | Low | Low | Unclear | Unclear | Low | Low | Low |
|  | **LOC-ED-Q and QEWP-C-5** | | | | | | |
| Altman et al. (2020)^a^ | Low | Low | Unclear | Unclear | Low | Low | Low |

*Abbreviations:* ADO-BED - Adolescent Binge-Eating Disorder Questionnaire; BEDS – Binge-Eating Disorder Screener; BEDT – Binge-Eating Disorder Test; BITE - Bulimic Inventory Test, Edinburgh; BULIT-R - Bulimia Test-Revised; CBBEQ - Children’s Brief Binge Eating Questionnaire; ChEDE-Q - Children’s Eating Disorder Examination-Questionnaire; CIA - Clinical Impairment Assessment; EDE-Q - Eating Disorder Examination Questionnaire; NEQ - Night Eating Questionnaire; QEWP - Questionnaire on Eating and Weight Patterns; QEWP-R - Questionnaire on Eating and Weight Patterns-Revised; LOC – loss of control; REO - Risk Factors for Binge-Eating Disorder in Overweight; YEDE-Q - Youth Eating Disorder Examination-Questionnaire

a. These studies evaluated more than one questionnaire and appear multiple times in the table.

**References**

Aardoom, J. J., Dingemans, A. E., Op't Landt, M. C. S., & Van Furth, E. F. (2012). Norms and discriminative validity of the Eating Disorder Examination Questionnaire (EDE-Q). *Eating Behaviors*, *13*(4), 305-309. https://doi.org/10.1016/j.eatbeh.2012.09.002

Allison, K. C., Lundgren, J. D., O'Reardon, J. P., Martino, N. S., Sarwer, D. B., Wadden, T. A., Crosby, R. D., Engel, S. G., & Stunkard, A. J. (2008). The Night Eating Questionnaire (NEQ): psychometric properties of a measure of severity of the Night Eating Syndrome. *Eating Behaviors*, *9*(1), 62-72. https://doi.org/10.1016/j.eatbeh.2007.03.007

Altman, D. R., Tanofsky-Kraff, M., Shank, L. M., Swanson, T. N., Ramirez, E., Moore, N. A., Rubin, S. G., Byrne, M. E., LeMay-Russell, S., Schvey, N. A., Kelly, N. R., Parker, M. N., Gubbi, S., Brady, S. M., Yanovski, S. Z., & Yanovski, J. A. (2020). Assessment of loss-of-control eating in healthy youth by interview and questionnaire. *International Journal of Eating Disorders*, *53*(5), 780-789. https://doi.org/10.1002/eat.23262

Bohn, K., Doll, H. A., Cooper, Z., O'Connor, M., Palmer, R. L., & Fairburn, C. G. (2008). The measurement of impairment due to eating disorder psychopathology. *Behaviour Research and Therapy*, *46*(10), 1105-1110. https://doi.org/10.1016/j.brat.2008.06.012

Borges, M. B. F., Morgan, C. M., Claudino, A. M., & Silveira, D. X. d. (2005). Validation of the Portuguese version of the Questionnaire on Eating and Weight Patterns: revised (QEWP-R) for the screening of binge eating disorder. *Brazilian Journal of Psychiatry*, *27*, 319-322. https://doi.org/10.1590/s1516-44462005000400012

Calugi, S., Pace, C. S., Muzi, S., Fasoli, D., Travagnin, F., & Dalle Grave, R. (2020). Psychometric proprieties of the Italian version of the questionnaire on eating and weight patterns (QEWP-5) and its accuracy in screening for binge-eating disorder in patients seeking treatment for obesity. *Eating and Weight Disorders*, *25*(6), 1739-1745. https://doi.org/10.1007/s40519-019-00818-1

Chamay-Weber, C., Combescure, C., Lanza, L., Carrard, I., & Haller, D. M. (2017). Screening Obese Adolescents for Binge Eating Disorder in Primary Care: The Adolescent Binge Eating Scale. *The Journal of Pediatrics*, *185*, 68-72.e61. https://doi.org/10.1016/j.jpeds.2017.02.038

Cooper, Z., & Fairburn, C. (1987). The eating disorder examination: A semi-structured interview for the assessment of the specific psychopathology of eating disorders. *International Journal of Eating Disorders*, *6*(1), 1-8. https://doi.org/10.1002/1098-108X(198701)6:1<1::AID-EAT2260060102>3.0.CO;2-9

de Man Lapidoth, J., Ghaderi, A., Halvarsson-Edlund, K., & Norring, C. (2007). Psychometric properties of the Eating Disorders in Obesity questionnaire: Validating against the Eating Disorder Examination interview. *Eating and Weight Disorders*, *12*(4), 168-175. https://doi.org/10.1007/BF03327594

De Zwaan, M., Mitchell, J. E., Specker, S. M., Pyle, R. L., Mussell, M. P., & Seim, H. C. (1993). Diagnosing binge eating disorder: Level of agreement between self‐report and expert‐rating. *International Journal of Eating Disorders*, *14*(3), 289-295. https://doi.org/10.1002/1098-108x(199311)14:3%3C289::aid-eat2260140307%3E3.0.co;2-0

Decaluwé, V., & Braet, C. (2004). Assessment of eating disorder psychopathology in obese children and adolescents: interview versus self-report questionnaire. *Behaviour Research and Therapy*, *42*(7), 799-811. https://doi.org/10.1016/j.brat.2003.07.008

Deeks, J., Bossuyt, P., Leeflang, M., & Takwoingi, Y. (Eds.). (2023). *Cochrane Handbook for Systematic Reviews of Diagnostic Test Accuracy* (2.0 ed.). Cochrane. https://training.cochrane.org/handbook-diagnostic-test-accuracy/current.

Deeks, J., Bossuyt, P. M., & Gatsonis, C. A. (Eds.). (2013). *Cochrane Handbook for Systematic Reviews of Diagnostic Test Accuracy*. The Cochrane Collaboration. https://methods.cochrane.org/sdt/handbook-dta-reviews.

Dymek-Valentine, M., Rienecke-Hoste, R., & Alverdy, J. (2004). Assessment of binge eating disorder in morbidly obese patients evaluated for gastric bypass: SCID versus QEWP-R. *Eating and Weight Disorders*, *9*(3), 211-216. https://doi.org/10.1007/bf03325069

Everett, V. S., Crochiere, R. J., Dallal, D. H., Martin, G. J., Manasse, S. M., & Forman, E. M. (2021). Self-report versus clinical interview: Discordance among measures of binge eating in a weight-loss seeking sample. *Eating and Weight Disorders*, *26(4)*, 1259-1263. https://doi.org/10.1007/s40519-020-01002-6

Fairburn, C. G., & Beglin, S. J. (1994). Assessment of eating disorders: interview or self-report questionnaire? *International Journal of Eating Disorders*, *16*(4), 363-370. https://doi.org/10.1002/1098-108X(199412)16:4%3C363::AID-EAT2260160405%3E3.0.CO;2-%23

Franklin, E. V., Simpson, V., Berthet-Miron, M., Gupta, O. T., & Barlow, S. E. (2019). A Pilot Study Evaluating a Binge-Eating Screener in Children: Development of the Children’s Brief Binge-Eating Questionnaire in a Pediatric Obesity Clinic. *Clinical Pediatrics*, *58*(10), 1063-1071. https://doi.org/10.1177/0009922819863664

Freitas, S. R., Lopes, C. S., Appolinario, J. C., & Coutinho, W. (2006). The assessment of binge eating disorder in obese women: a comparison of the binge eating scale with the structured clinical interview for the DSM-IV. *Eating Behaviors*, *7*(3), 282-289. https://doi.org/10.1016/j.eatbeh.2005.09.002

Ghaderi, A., & Scott, B. (2002). The preliminary reliability and validity of the Survey for Eating Disorders (SEDs): a self-report questionnaire for diagnosing eating disorders. *European Eating Disorders Review*, *10*(1), 61-76. https://doi.org/10.1002/erv.425

Goldschmidt, A. B., Doyle, A. C., & Wilfley, D. E. (2007). Assessment of binge eating in overweight youth using a questionnaire version of the Child Eating Disorder Examination with Instructions. *International Journal of Eating Disorders*, *40*(5), 460-467. https://doi.org/10.1002/eat.20387

Goossens, L., & Braet, C. (2010). Screening for eating pathology in the pediatric field. *International Journal of Pediatric Obesity*, *5*(6), 483-490. https://doi.org/10.3109/17477160903571995

Gormally, J., Black, S., Daston, S., & Rardin, D. (1982). The assessment of binge eating severity among obese persons. *Addictive Behaviors*, *7*(1), 47-55. https://doi.org/10.1016/0306-4603(82)90024-7

Gormez, A., Elbay, R. Y., Karatepe, H. T., Mutlu, H. H., & Karadere, M. E. (2023). Psychometric properties of the Turkish version of the Binge Eating Scale in detecting binge eating disorder among people seeking treatment for obesity. *Dusunen Adam - The Journal of Psychiatry and Neurological Sciences*, *36(4)*, 230-237. https://doi.org/10.14744/DAJPNS.2023.00229

Grupski, A. E., Hood, M. M., Hall, B. J., Azarbad, L., Fitzpatrick, S. L., & Corsica, J. A. (2013). Examining the Binge Eating Scale in screening for binge eating disorder in bariatric surgery candidates. *Obesity Surgery*, *23*(1), 1-6. https://doi.org/10.1007/s11695-011-0537-4

Hartmann, A. S., Gorman, M. J., Sogg, S., Lamont, E. M., Eddy, K. T., Becker, A. E., & Thomas, J. J. (2014). Screening for DSM-5 Other Specified Feeding or Eating Disorder in a Weight-Loss Treatment-Seeking Obese Sample. *The Primary Care Companion for CNS Disorders*, *16*(5). https://doi.org/10.4088/PCC.14m01665

Henderson, M., & Freeman, C. P. L. (1987). A Self-rating Scale for Bulimia the ‘BITE’. *British Journal of Psychiatry*, *150*(1), 18-24. https://doi.org/10.1192/bjp.150.1.18

Herman, B. K., Deal, L. S., DiBenedetti, D. B., Nelson, L., Fehnel, S. E., & Brown, T. M. (2016). Development of the 7-Item Binge-Eating Disorder Screener (BEDS-7). *The Primary Care Companion for CNS Disorders*, *18*(2). https://doi.org/10.4088/PCC.15m01896

House, E. T., Lister, N. B., Seidler, A. L., Li, H., Ong, W. Y., McMaster, C. M., Paxton, S. J., & Jebeile, H. (2022). Identifying eating disorders in adolescents and adults with overweight or obesity: A systematic review of screening questionnaires. *International Journal of Eating Disorders*, *55*(9), 1171-1193. https://doi.org/10.1002/eat.23769

Jeong, H., Hapenciuc, G., Meza, E., Le, J. T., Heinberg, L. J., & Marek, R. J. (2023). Reevaluating the Binge Eating Scale cut-off using DSM-5 criteria: analysis and replication in preoperative metabolic and bariatric surgery samples. *Surgery for Obesity and Related Diseases*, *19(9)*, 945-949. https://doi.org/10.1016/j.soard.2023.02.014

Johnson, W. G., Grieve, F. G., Adams, C. D., & Sandy, J. (1999). Measuring binge eating in adolescents: adolescent and parent versions of the questionnaire of eating and weight patterns. *International Journal of Eating Disorders*, *26*(3), 301-314. https://doi.org/10.1002/(sici)1098-108x(199911)26:3<301::aid-eat8>3.0.co;2-m

Kalarchian, M. A., Wilson, G. T., Brolin, R. E., & Bradley, L. (2000). Assessment of eating disorders in bariatric surgery candidates: Self‐report questionnaire versus interview. *International Journal of Eating Disorders*, *28*(4), 465-469. https://doi.org/10.1002/1098-108x(200012)28:4%3C465::aid-eat17%3E3.0.co;2-2

Liu, C.-Y., Tseng, M.-C. M., Chen, K.-Y., Chang, C.-H., Liao, S.-C., & Chen, H.-C. (2015). Sex difference in using the SCOFF questionnaire to identify eating disorder patients at a psychiatric outpatient clinic. *Comprehensive Psychiatry*, *57*, 160-166. https://doi.org/10.1016/j.comppsych.2014.11.014

Manasse, S. M., Michael, M. L., Lin, M., Gillikin, L., Zhang, F., Forman, E. M., & Juarascio, A. (2021). Can a Short Screening Tool Discriminate Between Overeating and Binge Eating in Treatment-Seeking Individuals with Obesity? *Obesity*, *29(4)*, 706-712. https://doi.org/10.1002/oby.23128

Mond, J. M., Myers, T. C., Crosby, R. D., Hay, P. J., Rodgers, B., Morgan, J. F., Lacey, J. H., & Mitchell, J. E. (2008). Screening for eating disorders in primary care: EDE-Q versus SCOFF. *Behaviour Research and Therapy*, *46*(5), 612-622. https://doi.org/10.1016/j.brat.2008.02.003

Morgan, J. F., Reid, F., & Lacey, J. H. (1999). The SCOFF questionnaire: assessment of a new screening tool for eating disorders. *BMJ*, *319*(7223), 1467-1468. https://doi.org/10.1136/bmj.319.7223.1467

Orlandi, E., Mannucci, E., Cuzzolaro, M., & SISDCA-Study Group on Psychometrics. (2005). Bulimic Investigatory Test, Edinburgh (BITE). A validation study of the Italian version. *Eating and Weight Disorders*, *10*(1), e14-e20. https://doi.org/10.1007/bf03354662

Parker, K., Mitchell, S., O’Brien, P., & Brennan, L. (2016). Psychometric evaluation of disordered eating measures in bariatric surgery candidates. *Obesity Surgery*, *26*(3), 563-575. https://doi.org/10.1007/s11695-015-1780-x

Quilliot, D., Brunaud, L., Mathieu, J., Quenot, C., Sirveaux, M.-A., Kahn, J.-P., Ziegler, O., & Witkowski, P. (2019). Links between traumatic experiences in childhood or early adulthood and lifetime binge eating disorder. *Psychiatry Research*, *276*, 134-141. https://doi.org/10.1016/j.psychres.2019.05.008

Ricca, V., Mannucci, E., Moretti, S., Di Bernardo, M., Zucchi, T., Cabras, P., & Rotella, C. (2000). Screening for binge eating disorder in obese outpatients. *Comprehensive Psychiatry*, *41*(2), 111-115. https://doi.org/10.1016/s0010-440x(00)90143-3

Shapiro, J. R., Woolson, S. L., Hamer, R. M., Kalarchian, M. A., Marcus, M. D., & Bulik, C. M. (2007). Evaluating binge eating disorder in children: Development of the children's binge eating disorder scale (C-BEDS). *International Journal of Eating Disorders*, *40*(1), 82-89. https://doi.org/10.1002/eat.20318

Solmi, F., Hatch, S. L., Hotopf, M., Treasure, J., & Micali, N. (2015). Validation of the SCOFF questionnaire for eating disorders in a multiethnic general population sample. *International Journal of Eating Disorders*, *48*(3), 312-316. https://doi.org/10.1002/eat.22373

Spitzer, R. L., Devlin, M., Walsh, B. T., Hasin, D., Wing, R., Marcus, M., Stunkard, A., Wadden, T., Yanovski, S., Agras, S., Mitchell, J., & Nonas, C. (1992). Binge eating disorder: A multisite field trial of the diagnostic criteria. *International Journal of Eating Disorders*, *11*(3), 191-203. https://doi.org/10.1002/1098-108X(199204)11:3<191::AID-EAT2260110302>3.0.CO;2-S

Thelen, M. H., Farmer, J., Wonderlich, S., & Smith, M. (1991). A revision of the Bulimia Test: The BULIT—R. *Psychological Assessment: A Journal of Consulting and Clinical Psychology*, *3*(1), 119. https://doi.org/10.1037/1040-3590.3.1.119

Vander Wal, J. S., Stein, R. I., & Blashill, A. J. (2011). The EDE-Q, BULIT-R, and BEDT as self-report measures of binge eating disorder. *Eating Behaviors*, *12*(4), 267-271. https://doi.org/10.1016/j.eatbeh.2011.07.006

Vander Wal, J. S., Waller, S. M., Klurfeld, D. M., McBurney, M. I., & Dhurandhar, N. V. (2005). Night eating syndrome: evaluation of two screening instruments. *Eating Behaviors*, *6*(1), 63-73. https://doi.org/10.1016/j.eatbeh.2004.04.004

Wadden, T. A., & Foster, G. D. (2006). Weight and Lifestyle Inventory (WALI). *Obesity*, *14*(S3), 99S-118S. https://doi.org/10.1038/oby.2006.289

Wever, M. C. M., Dingemans, A. E., Geerets, T., & Danner, U. N. (2018). Screening for Binge Eating Disorder in people with obesity. *Obesity Research & Clinical Practice*, *12*(3), 299-306. https://doi.org/10.1016/j.orcp.2018.02.002

Whiting, P., Rutjes, A., Westwood, M., Mallett, S., Deeks, J., Reitsma, J., Leelang, M., Sterne, J., & Bossuyt, P. (2011). QUADAS-2: A Revised Tool for the Quality Assessment of Diagnostic Accuracy Studies. *Annals of Internal Medicine*, *155*(8), 529-536. https://doi.org/10.7326/0003-4819-155-8-201110180-00009

Whiting, P., Rutjes, A. W., Reitsma, J. B., Bossuyt, P. M., & Kleijnen, J. (2003). The development of QUADAS: a tool for the quality assessment of studies of diagnostic accuracy included in systematic reviews. *BMC Medical Research Methodology*, *3*(1), 25. https://doi.org/10.1186/1471-2288-3-25

Yanovski, S. Z., Marcus, M. D., Wadden, T. A., & Walsh, B. T. (2015). The Questionnaire on Eating and Weight Patterns-5: An updated screening instrument for binge eating disorder. *International Journal of Eating Disorders*, *48*(3), 259-261. https://doi.org/10.1002/eat.22372
