# Supplementary material for: Identifying Eating Disorders in Adolescents and Adults Living With Higher Weight: An Updated Systematic Review of Questionnaire Diagnostic Accuracy
Source: Int J Eat Disord. 2026 May 20;59(9):1876–96. doi: 10.1002/eat.70132 (PMC13536217; doi:10.1002/eat.70132)
Supplement: Supplementary file 2 [file EAT-59-1876-s002.docx]

**Search Strategy: Medline** 2020 to 6^th^ November 2025

| **#** | **Searches** |
| --- | --- |
| 1 | Adolescent/ |
| 2 | exp Adult/ |
| 3 | adolescen*.tw. |
| 4 | adult*.tw. |
| 5 | youth*.tw. |
| 6 | child*.tw. |
| 7 | (young adj1 (people or person*)).tw. |
| 8 | p?ediatric*.tw. |
| 9 | m#n.tw. |
| 10 | wom#n.tw. |
| 11 | male*.tw. |
| 12 | female*.tw. |
| 13 | teen*.tw. |
| 14 | 1 or 2 or 3 or 4 or 5 or 6 or 7 or 8 or 9 or 10 or 11 or 12 or 13 |
| 15 | exp "Surveys and Questionnaires"/ |
| 16 | questionnaire*.tw. |
| 17 | survey*.tw. |
| 18 | screen*.tw. |
| 19 | exp Risk Assessment/ |
| 20 | 15 or 16 or 17 or 18 or 19 |
| 21 | (Diagnos* adj3 accurac*).tw. |
| 22 | agreement.tw. |
| 23 | validity.tw. |
| 24 | validation.tw. |
| 25 | "Sensitivity and Specificity"/ |
| 26 | sensitivity.tw. |
| 27 | specificity.tw. |
| 28 | 21 or 22 or 23 or 24 or 25 or 26 or 27 |
| 29 | exp "Feeding and Eating Disorders"/ |
| 30 | (disorder* adj4 eat*).tw. |
| 31 | anorex*.tw. |
| 32 | bulimi*.tw. |
| 33 | (bing* adj4 eat*).tw. |
| 34 | (diet* adj4 restrain*).tw. |
| 35 | night eating.tw. |
| 36 | pica.tw. |
| 37 | ruminat*.tw. |
| 38 | eating pathology.tw. |
| 39 | 29 or 30 or 31 or 32 or 33 or 34 or 35 or 36 or 37 or 38 |
| 40 | 14 and 20 and 28 and 39 |
| 41 | limit 40 to yr="2020 -Current" |

Database(s): **Embase Classic+Embase** 2020 to 6^th^ November 2025 
Search Strategy:

| **#** | **Searches** |
| --- | --- |
| 1 | exp adolescent/ |
| 2 | exp adult/ |
| 3 | adolescen*.tw. |
| 4 | adult*.tw. |
| 5 | youth*.tw. |
| 6 | child*.tw. |
| 7 | (young adj1 (people or person*)).tw. |
| 8 | p?ediatric*.tw. |
| 9 | m#n.tw. |
| 10 | wom#n.tw. |
| 11 | male*.tw. |
| 12 | female*.tw. |
| 13 | teen*.tw. |
| 14 | 1 or 2 or 3 or 4 or 5 or 6 or 7 or 8 or 9 or 10 or 11 or 12 or 13 |
| 15 | exp questionnaire/ |
| 16 | questionnaire*.tw. |
| 17 | survey*.tw. |
| 18 | screen*.tw. |
| 19 | exp risk assessment/ |
| 20 | 15 or 16 or 17 or 18 or 19 |
| 21 | (diagnos* adj3 accurac*).tw. |
| 22 | agreement.tw. |
| 23 | validity.tw. |
| 24 | validation.tw. |
| 25 | exp "sensitivity and specificity"/ |
| 26 | sensitivity.tw. |
| 27 | specificity.tw. |
| 28 | 21 or 22 or 23 or 24 or 25 or 26 or 27 |
| 29 | exp eating disorder/ |
| 30 | (disorder* adj4 eat*).tw. |
| 31 | anorex*.tw. |
| 32 | bulimi*.tw. |
| 33 | (bing* adj4 eat*).tw. |
| 34 | (diet* adj4 restrain*).tw. |
| 35 | night eating.tw. |
| 36 | pica.tw. |
| 37 | ruminat*.tw. |
| 38 | eating pathology.tw. |
| 39 | 29 or 30 or 31 or 32 or 33 or 34 or 35 or 36 or 37 or 38 |
| 40 | 14 and 20 and 28 and 39 |
| 41 | limit 40 to yr="2020 -Current" |

**Database: CINAHL**

**Search strategy:** 1^st^ September 2020 to 7^th^ November 2025

| S15 | S4 AND S8 AND S11 AND S14 | Limiters - Publication Date: 20200901-20251131 Expanders - Apply equivalent subjects Search modes - Proximity | Interface - EBSCOhost Research Databases Search Screen - Advanced Search Database - CINAHL Complete |
| --- | --- | --- | --- |
| S14 | S12 OR S13 | Expanders - Apply equivalent subjects Search modes - Proximity | Interface - EBSCOhost Research Databases Search Screen - Advanced Search Database - CINAHL Complete |
| S13 | TI ((disorder* N4 eat*) OR anorex* OR bulmi* OR (bing* N4 eat*) OR (diet* N4 restrain*) OR "night eating" OR pica OR ruminat* OR "eating pathology") OR AB ((disorder* N4 eat*) OR anorex* OR bulmi* OR (bing* N4 eat*) OR (diet* N4 restrain*) OR "night eating" OR pica OR ruminat* OR "eating pathology") | Expanders - Apply equivalent subjects Search modes - Proximity | Interface - EBSCOhost Research Databases Search Screen - Advanced Search Database - CINAHL Complete |
| S12 | (MH "Eating Disorders+") OR (MH "Binge Eating Disorder") OR (MH "Avoidant Restrictive Food Intake Disorder") OR (MH "Bulimia Nervosa") | Expanders - Apply equivalent subjects Search modes - Proximity | Interface - EBSCOhost Research Databases Search Screen - Advanced Search Database - CINAHL Complete |
| S11 | S9 OR S10 | Expanders - Apply equivalent subjects Search modes - Proximity | Interface - EBSCOhost Research Databases Search Screen - Advanced Search Database - CINAHL Complete |
| S10 | TI (validity OR validation OR sensitivity OR specificity OR (diagnos* N3 accurac*) OR agreement) OR AB (validity OR validation OR sensitivity OR specificity OR (diagnos* N3 accurac*) OR agreement) | Expanders - Apply equivalent subjects Search modes - Proximity | Interface - EBSCOhost Research Databases Search Screen - Advanced Search Database - CINAHL Complete |
| S9 | (MH "Sensitivity and Specificity") | Expanders - Apply equivalent subjects Search modes - Proximity | Interface - EBSCOhost Research Databases Search Screen - Advanced Search Database - CINAHL Complete |
| S8 | S5 OR S6 OR S7 | Expanders - Apply equivalent subjects Search modes - Proximity | Interface - EBSCOhost Research Databases Search Screen - Advanced Search Database - CINAHL Complete |
| S7 | TI (questionnaire* OR survey* OR screen* OR "risk assessment*") OR AB (questionnaire* OR survey* OR screen* OR "risk assessment*") | Expanders - Apply equivalent subjects Search modes - Proximity | Interface - EBSCOhost Research Databases Search Screen - Advanced Search Database - CINAHL Complete |
| S6 | (MH "Surveys+") | Expanders - Apply equivalent subjects Search modes - Proximity | Interface - EBSCOhost Research Databases Search Screen - Advanced Search Database - CINAHL Complete |
| S5 | (MH "Questionnaires+") | Expanders - Apply equivalent subjects Search modes - Proximity | Interface - EBSCOhost Research Databases Search Screen - Advanced Search Database - CINAHL Complete |
| S4 | S1 OR S2 OR S3 | Expanders - Apply equivalent subjects Search modes - Proximity | Interface - EBSCOhost Research Databases Search Screen - Advanced Search Database - CINAHL Complete |
| S3 | TI (adolescen* OR adult* OR youth* OR child* OR (young N1 (people OR person*)) OR p#ediatric* OR man OR men OR woman OR women OR male* OR female* OR teen*) OR AB (adolescen* OR adult* OR youth* OR child* OR (young N1 (people OR person*)) OR p#ediatric* OR man OR men OR woman OR women OR male* OR female* OR teen*) | Expanders - Apply equivalent subjects Search modes - Proximity | Interface - EBSCOhost Research Databases Search Screen - Advanced Search Database - CINAHL Complete |
| S2 | (MH "Adult+") | Expanders - Apply equivalent subjects Search modes - Proximity | Interface - EBSCOhost Research Databases Search Screen - Advanced Search Database - CINAHL Complete |
| S1 | (MH "Adolescence") | Expanders - Apply equivalent subjects Search modes - Proximity | Interface - EBSCOhost Research Databases Search Screen - Advanced Search Database - CINAHL Complete |

Database(s): **APA PsycInfo** 2020 to 6^th^ November 2025
Search Strategy:

| **#** | **Searches** |
| --- | --- |
| 1 | adolescen*.tw. |
| 2 | adult*.tw. |
| 3 | youth*.tw. |
| 4 | child*.tw. |
| 5 | (young adj1 (people or person*)).tw. |
| 6 | p?ediatric*.tw. |
| 7 | m#n.tw. |
| 8 | wom#n.tw. |
| 9 | male*.tw. |
| 10 | female*.tw. |
| 11 | teen*.tw. |
| 12 | 1 or 2 or 3 or 4 or 5 or 6 or 7 or 8 or 9 or 10 or 11 |
| 13 | exp Surveys/ |
| 14 | exp Questionnaires/ |
| 15 | questionnaire*.tw. |
| 16 | survey*.tw. |
| 17 | screen*.tw. |
| 18 | exp Risk Assessment/ |
| 19 | 13 or 14 or 15 or 16 or 17 or 18 |
| 20 | (Diagnos* adj3 accurac*).tw. |
| 21 | agreement.tw. |
| 22 | validity.tw. |
| 23 | validation.tw. |
| 24 | exp Test Sensitivity/ |
| 25 | exp Test Specificity/ |
| 26 | sensitivity.tw. |
| 27 | specificity.tw. |
| 28 | 20 or 21 or 22 or 23 or 24 or 25 or 26 or 27 |
| 29 | exp eating disorders/ |
| 30 | (disorder* adj4 eat*).tw. |
| 31 | anorex*.tw. |
| 32 | bulimi*.tw. |
| 33 | (bing* adj4 eat*).tw. |
| 34 | (diet* adj4 restrain*).tw. |
| 35 | night eating.tw. |
| 36 | pica.tw. |
| 37 | ruminat*.tw. |
| 38 | eating pathology.tw. |
| 39 | 29 or 30 or 31 or 32 or 33 or 34 or 35 or 36 or 37 or 38 |
| 40 | 12 and 19 and 28 and 39 |
| 41 | limit 40 to yr="2020 -Current" |

Database(s): **APA PsycTests** 2020 to 7^th^ November 2025 
Search Strategy:

| **#** | **Searches** |
| --- | --- |
| 1 | exp Adolescent Psychiatry/ or exp Adolescent Psychology/ or exp Adolescent Health/ |
| 2 | adult*.tw. |
| 3 | adolescen*.tw. |
| 4 | youth.tw. |
| 5 | child*.tw. |
| 6 | (young adj1 (people or person*)).tw. |
| 7 | p?ediatric*.tw. |
| 8 | m#n.tw. |
| 9 | wom#n.tw. |
| 10 | male*.tw. |
| 11 | female*.tw. |
| 12 | teen*.tw. |
| 13 | 1 or 2 or 3 or 4 or 5 or 6 or 7 or 8 or 9 or 10 or 11 or 12 |
| 14 | exp surveys/ |
| 15 | exp questionnaires/ |
| 16 | questionnaire*.tw. |
| 17 | survey*.tw. |
| 18 | screen*.tw. |
| 19 | exp risk assessment/ |
| 20 | 14 or 15 or 16 or 17 or 18 or 19 |
| 21 | (diagnos* adj3 accurac*).tw. |
| 22 | agreement.tw. |
| 23 | validity.tw. |
| 24 | validation.tw. |
| 25 | exp test sensitivity/ |
| 26 | exp test specificity/ |
| 27 | sensitivity.tw. |
| 28 | specificity.tw. |
| 29 | 21 or 22 or 23 or 24 or 25 or 26 or 27 or 28 |
| 30 | exp eating disorders/ |
| 31 | (disorder* adj4 eat*).tw. |
| 32 | anorex*.tw. |
| 33 | bulimi*.tw. |
| 34 | (bing* adj4 eat*).tw. |
| 35 | (diet* adj4 restrain*).tw. |
| 36 | night eating.tw. |
| 37 | pica.tw. |
| 38 | ruminat*.tw. |
| 39 | eating pathology.tw. |
| 40 | 30 or 31 or 32 or 33 or 34 or 35 or 36 or 37 or 38 or 39 |
| 41 | 13 and 20 and 29 and 40 |
| 42 | limit 41 to yr="2020 -Current" |
